# A highly stable and hierarchical tetrathiafulvalene-based metal–organic framework with improved performance as a solid catalyst†
†Electronic supplementary information (ESI) available. CCDC 1579606 (**MUV-2**). For ESI and crystallographic data in CIF or other electronic format see DOI: 10.1039/c7sc04829g


**DOI:** 10.1039/c7sc04829g

**Published:** 2018-01-24

**Authors:** Manuel Souto, Andrea Santiago-Portillo, Miguel Palomino, Iñigo J. Vitórica-Yrezábal, Bruno J. C. Vieira, João C. Waerenborgh, Susana Valencia, Sergio Navalón, Fernando Rey, Hermenegildo García, Guillermo Mínguez Espallargas

**Affiliations:** a Instituto de Ciencia Molecular (ICMol) , Universitat de València , c/Catedrático José Beltrán, 2 , 46980 Paterna , Spain . Email: guillermo.minguez@uv.es; b Departamento de Química , Universitat Politècnica de València , c/Camino de Vera, s/n , 46022 , Valencia , Spain; c Instituto de Tecnología Química (UPV-CSIC) , Universitat Politècnica de València-Consejo Superior de Investigaciones Científicas , Av. De los Naranjos s/n , 46022 , Valencia , Spain; d School of Chemistry , University of Manchester , Oxford Road , Manchester , M139PL , UK; e Centro de Ciências e Tecnologias Nucleares , Instituto Superior Técnico , Universidade de Lisboa , 2695-066 Bobadela LRS , Portugal

## Abstract

Herein we report the synthesis of a tetrathiafulvalene (TTF)-based MOF, namely **MUV-2**, which shows a non-interpenetrated hierarchical crystal structure with mesoporous one-dimensional channels of *ca.* 3 nm and orthogonal microporous channels of *ca.* 1 nm. This highly stable MOF (aqueous solution with pH values ranging from 2 to 11 and different organic solvents), which possesses the well-known [Fe_3_(μ_3_-O)(COO)_6_] secondary building unit, has proven to be an efficient catalyst for the aerobic oxidation of dibenzothiophenes.

## Abstract 2

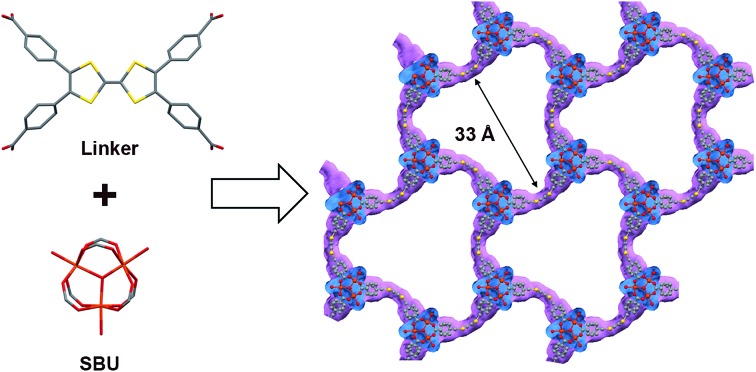
A highly stable Metal–Organic Framework with a hierarchical structure based on the Fe_3_O cluster and a TTF-based ligand is presented.

## Introduction

During the last two decades, the design and preparation of metal–organic frameworks (MOFs)^1^–^3^ have attracted a great deal of attention due to their high potential in several applications such gas storage and separation,^4^ sensing,^5^ and catalysis,^6^ among others. In particular, the combination of both large pores and high stability is of high interest towards practical applications. However, most reported MOFs are microporous (pore size < 2 nm) and there are only a few examples of mesoporous MOFs (2–50 nm) that combine both large pore sizes and high stability such as MIL-100, MIL-101 or PCN-600.^7^–^9^ Recently, the preparation of microporous-mesoporous hierarchical MOFs has become a subject of great interest since micropores contribute to the bulk of the surface area, whereas mesopores provide better accessibility to larger molecules to diffuse quickly, becoming very attractive for catalytic applications.^10^ During the past few years, several strategies have been reported to construct hierarchical MOFs that usually require multi-step and lengthy synthetic procedures which also lack structural control of the mesopores.^11^–^14^ In contrast, the direct formation of highly stable and hierarchical MOFs presenting both micro- and mesopores in their crystalline structures (*i.e.* in an ordered manner) is limited, to our knowledge, to the material NU-1000, which is based on the Zr_6_(μ_3_-OH)_8_(OH)_8_ cluster and 1,3,6,8-tetrakis(*p*-benzoic acid)pyrene ligand.^15^ This material possesses micropores and mesopores that run parallel along the same axis direction. In catalysis, it has been found that the combination of micro- and mesopores in a hierarchical material increases its activity notably by favoring the diffusion of substrates and reagents, particularly bulky reagents.^16^

On the other hand, tetrathiafulvalene (TTF) and its numerous derivatives are among the most versatile molecules which exhibit interesting redox properties, electron–donor character and potential application as molecular conductors.^17^ The use of TTF as a ligand for the design of porous coordination polymers can give rise to multifunctional materials combining different physical properties.^18^–^20^ For example, Dincă and coworkers have recently reported the use of the ligand tetrathiafulvalene tetrabenzoic acid (H_4_TTFTB) with various transition metals (II) obtaining a family of isostructural and microporous TTF-based MOFs exhibiting tunable electrical conductivity.^21^,^22^ More recently, the same ligand has also been used for the preparation of a TTF-based MOF with Mg(ii), which exhibits permanent mesopores.^23^

Herein, we report the synthesis, structure determination and physical properties of **MUV-2** (MUV: Materials of the University of Valencia), a highly stable TTF-based MOF with a unique non-interpenetrated hierarchical crystal structure with the mesoporous channels orthogonal to the microporous channels. Moreover, the advantages of **MUV-2** with respect to widely used MOF catalysts will be clearly demonstrated for a reaction of great interest in the field, illustrating the advantages of having a hierarchical MOF with large mesopores and high stability.

## Results and discussion

**Scheme 1:**
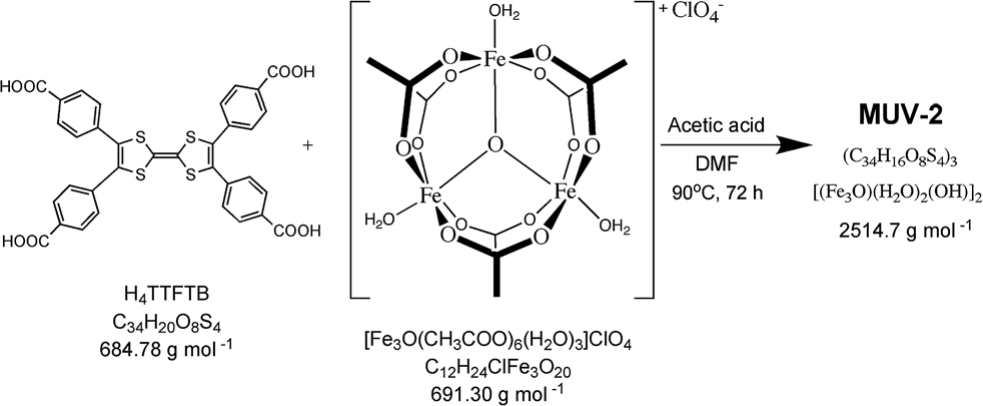
Synthesis of **MUV-2**.

**MUV-2** was prepared according to an adapted synthetic methodology^24^ using the preformed cluster [Fe_3_O(CH_3_COO)_6_]ClO_4_ as the starting material. The reaction of [Fe_3_O(CH_3_COO)_6_]ClO_4_, H_4_TTFTB, and acetic acid in *N*,*N*-dimethylformamide (DMF) at 90 °C for 72 h yielded dark red needle-like single crystals of **MUV-2** that were used to determine the crystal structure by single-crystal X-ray diffraction (XRD) (Scheme 1). The bulk material was exhaustively washed with a large amount of DMF and immersed in the solvent overnight in order to remove any unreacted starting materials. Finally, the powder was immersed in EtOH at 65 °C for 3 h, filtered and dried at room temperature. The infrared (IR) spectra of the powder confirmed that there was no unreacted starting material present (Fig. S1†).

**Fig. 1:**
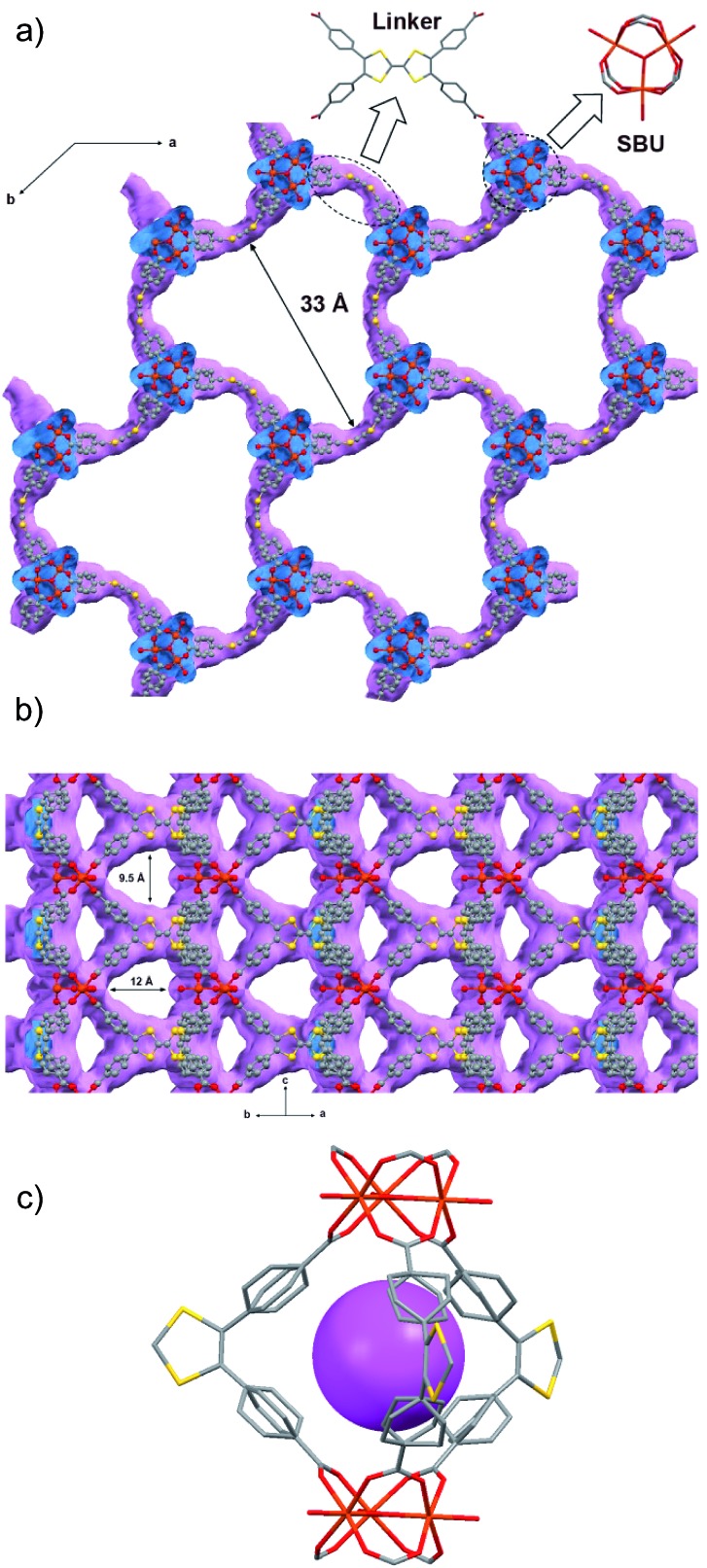
(a) Representation of the crystal structure of **MUV-2** showing mesoporous channels along the *c*-axis and (b) microporous channels orthogonal to the *c*-axis. The van der Waals surface is shown in purple. (c) Microporous cage formed by three TTFTB ligands and two [Fe_3_(μ_3_-O)(COO)_6_] SBUs, with the purple ball placed in the structure to represent the void. The grey, yellow, red and orange ellipsoids represent the C, S, O and Fe atoms, respectively. For simplicity, hydrogens are omitted.

Single-crystal X-ray diffraction data were collected with up to 1 Å resolution at the I19 beamline facilities at the Diamond Light Source (UK). **MUV-2** crystallises in the space group *P*62*m* and the unit cell parameters are *a* = *b* = 33.3 Å and *c* = 12.4 Å and it consists of 6-connected [Fe_3_(μ_3_-O)(COO)_6_] SBUs and tetratopic TTFTB ligands. Considering each TTFTB ligand as a four-connected node and each Fe_3_O(COO)_6_ unit as a six-connected node, **MUV-2** can be simplified as a 4,6-connected network with ttp topology (Fig. S2†), an unusual topology previously observed in two lanthanoid-based MOFs.^25^,^26^ The non-interpenetrated crystal structure reveals large hexagonal mesoporous 1-D channels of *ca.* 3 nm along the *c*-axis, which are formed by six TTFTB ligands and six clusters [Fe_3_(μ_3_-O)(COO)_6_] (Fig. 1a). TTF moieties are significantly twisted around the central C=C bond with a dihedral angle of 20°, whereas the planes formed by the two dithiole rings (planes S1–C1–C2–S2 and S3–C5–C6–S4) have a dihedral angle of 41° (Fig. S3†). The torsion angles of S2–C3–S1–C1 and C1–C2–S2–C3 are 17° and 11°, respectively, which are typical for neutral TTFs. The phenyl rings exhibit large distortions with respect to the TTF core with dihedral angles of 62° with the latter. In contrast to NU-1000, where the microporous channels run parallel to the mesoporous ones,^15^ the crystal structure of **MUV-2** shows that microporous channels of *ca.* 1 nm (9.5 × 12 Å) are orthogonal to the mesoporous channels and are formed between two TTFTB ligands and two [Fe_3_(μ_3_-O)(COO)_6_] clusters (Fig. 1b). In addition, microporous cages consist of three TTFTB ligands and two [Fe_3_(μ_3_-O)(COO)_6_] SBUs (Fig. 1c) leading to a remarkable open structure with a calculated free volume of *ca.* 82%. Note that the crystal structure of **MUV-2** contains the precursor [Fe_3_O(CH_3_COO)_6_]ClO_4_ within the pores since it was determined from the as-synthesised material without the washing and activation procedure.

The results obtained from Mössbauer spectroscopy, magnetic measurements, solid-state cyclic voltammetry and Raman spectroscopy are consistent with the [Fe_3_(μ_3_-O)(COO)_6_] cluster being formed by three *S* = 5/2 Fe(iii) ions in octahedral environments, and the TTF cores being neutral, thus yielding a material with the formula (TTFTB)_3_[(Fe_3_O)(H_2_O)_2_(OH)]_2_, which is in agreement with the EDAX analysis of **MUV-2** (see Fig. S4–S9†). It is important to note that one of the three coordinated H_2_O molecules in the cluster is present as a negatively charged hydroxide (OH^–^) in order to maintain the charge balance.

**Fig. 2:**
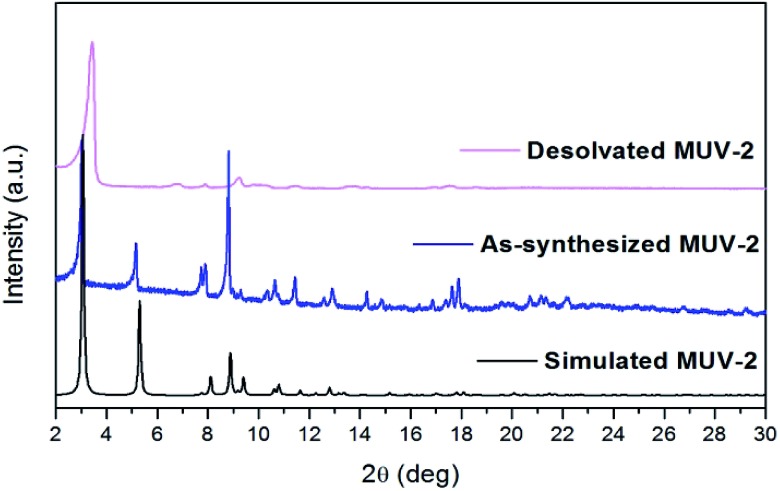
Powder X-ray diffraction patterns of simulated, as-synthesised and desolvated **MUV-2**.

Thermogravimetric analysis (TGA) of washed **MUV-2** exhibited a sharp mass loss of 20% between 25 and 100 °C, which corresponds to the elimination of solvent molecules (Fig. S10†). TGA shows a large plateau above 200 °C until the final decomposition at 350 °C. Activation of **MUV-2** was performed by heating the washed material at 150 °C for 2 h. Its crystallinity was confirmed by powder X-ray diffraction (PXRD) and it was observed that the principal peak was slightly shifted to 3.4° upon heating (Fig. 2) and recovered to the initial PXRD pattern upon resolvation (Fig. S11†). Additionally, **MUV-2** shows extraordinary chemical stability in aqueous solution with pH values ranging from 2 to 11 and in different organic solvents for 24 h. The PXRD patterns showed that crystallinity is maintained under these conditions (Fig. S12†) and the CO_2_ adsorption capacity is well preserved, for example, after treatment with pH = 2 and 11 aqueous solutions (Fig. S20†) with only a minor reduction.

**Fig. 3:**
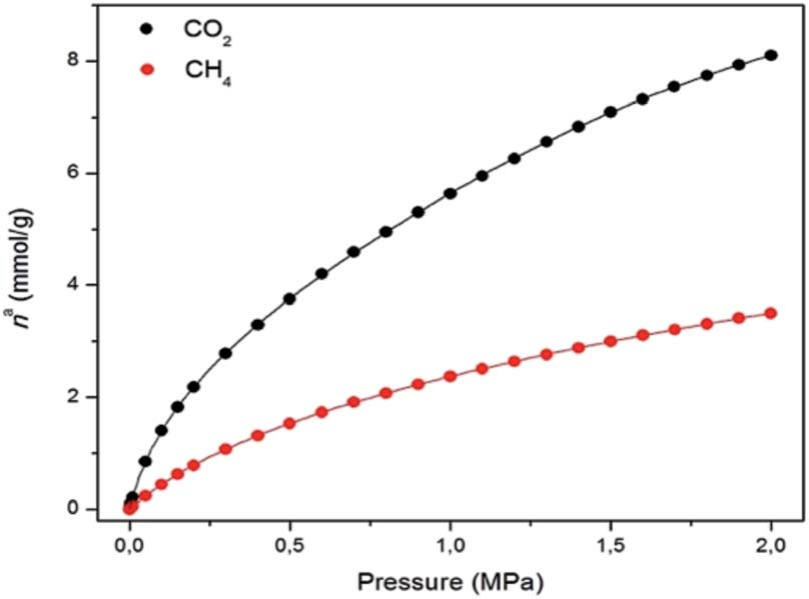
Gas adsorption isotherms of CO_2_ (black) and CH_4_ (red) on **MUV-2** at 298 K (lines correspond to the best fits). Data at other temperatures are shown in the ESI.†

The N_2_ adsorption isotherm at 77 K revealed a combination of Type I and IV isotherms, resulting from the presence of micropores and mesopores, respectively. Thus, a steep N_2_ adsorption occurs at low *p*/*p*_0_, while a slight secondary uptake was also found due to the mesopores filling. A plateau was observed in the N_2_ uptake of 16 mmol g^–1^ (Fig. S13†). **MUV-2** has a BET surface area of 1220 m^2^ g^–1^, which is higher than those for other reported mesoporous TTF-based MOFs.^23^ A micropore volume of 0.52 cm^3^ g^–1^ was found using the Dubinin–Radushkevich equation and the pore size analysis by means of the Barrett–Joyner–Halenda (BJH) method revealed a pore size of 38.7 Å (Fig. S15†). Fig. 3 shows the CO_2_ and CH_4_ isotherms at 298 K, revealing a high sorption capacity for both gases comparable to that of MIL-100.^27^

The isosteric heat of adsorption (*q*_st_) of CO_2_ decreases from 30 to 20 kJ mol^–1^, and remains constant in the case of CH_4_ at around 16 kJ mol^–1^ within the studied loading range (Fig. S18†). These values clearly indicate the higher affinity of **MUV-2** for CO_2_ than for CH_4_. The isosteric heat of adsorption of CO_2_ at zero coverage (*q*0st) of **MUV-2** is comparable to that of an LTA zeolite with a Si/Al ratio of around 6,^28^ and to that of MIL-101, and is in the same range as a wide variety of MOFs.^29^

**Scheme 2:**
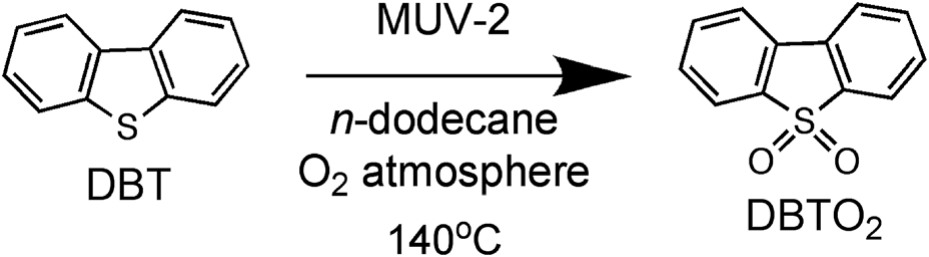
Aerobic oxidation of DBT to DBTO_2_ using **MUV-2** as a catalyst.

The superior catalytic activity of **MUV-2** due to the presence of mesopores with respect to widely used MIL MOFs as heterogeneous catalysts was clearly evidenced for the aerobic oxidation of dibenzothiophene (DBT) using long chain alkanes as solvents (Scheme 2). DBT is a model compound of the harmful aromatic sulphur compounds present in diesel.^16^ Legal regulations require diminishing the sulphur content in diesel down to the ppb scale. One possibility is to perform fuel oxidation to convert the sulphur-containing organic compounds to the corresponding sulfones (generally soluble in water) that can be removed from the fuel by washing. It has recently been reported that DBT can be oxidized by molecular oxygen to the corresponding sulfone (DBTO_2_) using MIL-101(Cr or Fe) as the solid catalyst,^16^ although an induction period, probably related to diffusion problems, was observed.

**Fig. 4:**
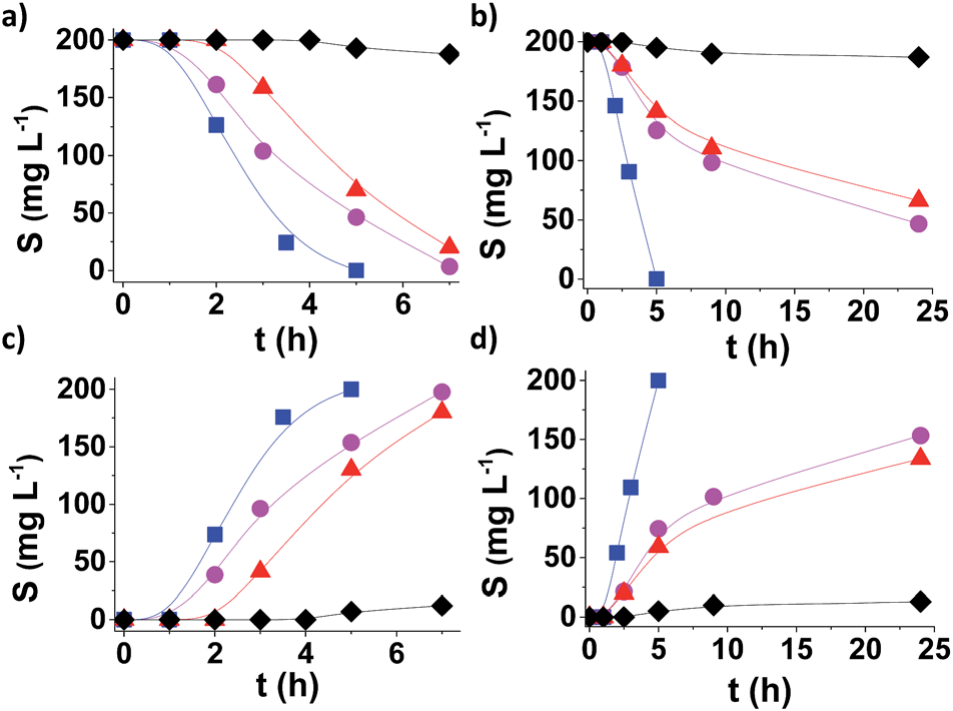
Aerobic oxidation of DBT (a and b) to DBTO_2_ (c and d), plotted as sulfur content, using **MUV-2** (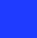), MIL-101 (Fe) (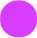), and MIL-100 (Fe) (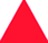) and in the absence of a catalyst (♦) using *n*-dodecane (a and c) or diesel (b and d). Reaction conditions: 0.012 mmol of Fe catalyst, DBT (1150 mg L^–1^), solvent (10 mL), O_2_ (1 atm) and 140 °C.

Fig. 4 shows the time conversion plots for DBT disappearance and DBTO_2_ formation in *n*-dodecane (plotted as sulphur content) comparing the temporal profile using **MUV-2**, MIL-101 (Fe) and MIL-100 (Fe), and it shows that **MUV-2** is the best performing catalyst. Since all three MOFs contained the same type of Fe_3_-μ_3_O active centre, the higher catalytic activity of **MUV-2** can be attributed to the more favourable diffusion due to the presence of large pores in this material (see Table S3†), as demonstrated with three different control experiments. To gain understanding on the origin of the induction period and its dependency on the pore size of **MUV-2**, this solid was contacted with *n*-dodecane containing DBT in the absence of O_2_ for 2 h, and then O_2_ was introduced into the flask, whereby an immediate oxidation of DBT without an induction period was observed (see Fig. S21a†). A similar observation, *i.e.* a lack of induction period, was also noted when **MUV-2** was boiled in *n*-dodecane containing O_2_ and DBT was added two hours later (see Fig. S21b†). Finally, no reduction of the induction period is observed if **MUV-2** is heated in *n*-dodecane for two hours before introducing O_2_ and DBT to the system (see Fig. S21c†). This rationalisation is in agreement with the fact that, besides higher reaction rates, the induction period is remarkably shortened to about 1 h using **MUV-2**.

From an application point of view, desulfuration of diesel should be carried out in the presence of a mixture of hydrocarbons from C13 to C18. Since diffusion is a limiting factor under this condition, the changes from model *n*-dodecane as the solvent to real diesel were accompanied by a considerable decrease in activity in the case of MIL-101.^16^ It is of interest, therefore, to determine the performance of **MUV-2** for DBT aerobic oxidation using diesel as the solvent. The results presented in Fig. 4b and d provide a comparison of the time–conversion plots for DBT oxidation using MIL-101 (Fe), MIL-100 (Fe) and **MUV-2**. As is observed, the difference in catalytic activity when using diesel as the solvent remarkably favors **MUV-2**, and shows the advantages of this MOF under these conditions. Reusability and productivity tests also show the high stability of **MUV-2** as a solid catalyst (Fig. S22 and S23†), which is also active for different DBT derivatives (4-MeDBT and 4,6-Me_2_DBT) (Fig. S24†).

Hot-filtration tests were performed by filtering **MUV-2** out from the hot reaction mixture after 2 h. At this point, the sulphur content was about 125 ppm (*cf.* the initial 200 mg L^–1^), and the clear supernatant was allowed to continue to react in the absence of solid particles, and we observed a very minor progress of about 20 mg L^–1^ sulphur content decrease in the subsequent 3 h (Fig. S25c†). In contrast, a twin reaction where **MUV-2** was not filtered achieved complete sulphur removal from the initial 200 mg L^–1^ in 5 h. These results indicate that after initiation of the reaction only a very minor contribution of leaching and homogeneous oxidation is present. In addition, control experiments that used either chromium(iii) acetate (0.6 mg of Cr), the preformed [Fe_3_O(CH_3_COO)_6_]ClO_4_ at the loadings corresponding to those present in **MUV-2** or leached out during the reaction, or H_4_TTFTB as homogeneous catalysts showed negligible conversion in all cases, indicating that Cr and Fe transition metals at these concentrations and the ligand are not able to promote DBT oxidation (Fig. S25†).

A combination of quenching experiments and spectroscopic studies has been used to address the reaction mechanism and, in particular, to determine that the primary reactive oxygen species responsible for oxidation is HOO˙ (see Fig. S26 and S27†). Thus, performing the oxidation in the presence of DMSO, a selective hydroxyl radical scavenger, does not influence the time–conversion plot much, while, in contrast, the presence of *p*-benzoquinone, a selective quencher of superoxide and hydroperoxyl radicals, strongly inhibits DBT oxidation to DBTO_2_. In addition, admission of oxygen into thermally dehydrated **MUV-2** (220 °C, 5 h) at 140 °C led to the appearance of two new vibration bands in the Raman spectra at 1502 and 1161 cm^–1^ that can be attributed to physisorbed O_2_ and Fe–O–O, respectively (Fig. S27†). This metal-peroxo could abstract a hydrogen atom from the medium (*n*-dodecane), generating hydroperoxyl radicals that would initiate DBT oxidation. This hypothesis is supported by the observation of some very minor undetermined oxidation products from *n*-dodecane.

## Conclusions

In summary, we have demonstrated that **MUV-2**, which possesses a hierarchical crystal structure with hexagonal mesoporous channels running orthogonal to the micropores, shows high thermal and chemical stability. This hierarchical structure is highly relevant for the catalytic activity of **MUV-2** in the aerobic oxidation of DBT in long chain alkanes as solvents, whereby a dramatic increase in activity with respect to related MIL-100 and MIL-101 catalysts has been observed.

## Experimental

### General methods and materials

All reagents and solvents employed for the syntheses were of high purity grade and were purchased from Sigma-Aldrich Co. and TCI. ^1^H NMR spectra were recorded using a Bruker DPX300 (300 MHz) spectrometer and Me_4_Si as an internal standard. Infrared spectra were recorded using a FT-IR Nicolet 5700 spectrometer in the 4000–400 cm^–1^ range using powdered samples diluted in KBr pellets. Thermogravimetric analysis was carried out using a Mettler Toledo TGA/SDTA 851 apparatus in the 25–600 °C temperature range with a 10 °C min^–1^ scan rate and a N_2_ flow of 30 mL min^–1^. Powder X-ray diffraction spectra were recorded using 0.7 mm borosilicate capillaries that were aligned on an Empyrean PANalytical powder diffractometer, using Cu Kα radiation (*λ* = 1.54056 Å).

### Synthesis of **MUV-2**

Bulk: 20 mg of H_4_TTFTB, 20 mg of [Fe_3_O(CH_3_COO)_6_]ClO_4_ and 0.8 mL of acetic acid were dissolved in 4 mL of DMF in a 10 mL Pyrex vial. The mixture was heated in an oven at 90 °C for 72 h. After cooling down to room temperature, a dark brown powder was collected by filtration. The powder was washed with a large amount of DMF in order to remove the unreacted ligand and [Fe_3_O(CH_3_COO)_6_]ClO_4_ and was immersed in DMF overnight. Finally, the product was immersed in EtOH for 3 h at 65 °C, and was then washed and collected by filtration (19.6 mg; 80%).

### Crystal data for **MUV-2**

X-ray data was collected at 100 K for the red needle-like crystals with synchrotron radiation using a single crystal X-ray diffraction beamline I19 at the Diamond Light Source, equipped with a Pilatus 2M detector and an Oxford Cryosystems nitrogen flow gas system. Despite using synchrotron radiation, **MUV-2** crystals only diffracted to a resolution of 1.2 Å. Refinement details can be found in the ESI.† Space group *P*62*m*, *a* = *b* = 33.298(3) Å, *c* = 12.3958(7) Å, *V* = 11 903(2) Å^3^, *R*_1_ (F) = 0.0324, w*R*_2_ (*F*^2^) = 0.0929.

### Gas sorption

High-pressure adsorption isotherms of CO_2_, CH_4_ and N_2_ were measured at different temperatures ranging from 283 to 333 K in an IGA-3 gravimetric analyser (Hiden Isochema) using approximately 50 mg of sample in the balance. Before each adsorption experiment, the sample was outgassed at 423 K under a final pressure of 10^–5^ Pa for four hours. The sample was then cooled down under high vacuum to the target temperature that was controlled using a recirculating thermostatic bath. Adsorption measurements were performed by introducing the gas to build up the desired pressures of the isotherms. The heat of adsorption was calculated according to the Clausius–Clapeyron equation from the isotherms measured at different temperatures.

### Magnetic measurements

Magnetic susceptibility measurements were carried out on single-phase polycrystalline samples with a Quantum Design MPMS-XL-5 SQUID susceptometer. The susceptibility data were all collected at 1 K min^–1^, in the range 2–300 K with an applied field of 0.1 T. The susceptibility data were corrected from the diamagnetic contributions as deduced using Pascal’s constant tables.

### Mössbauer spectroscopy measurements

Mössbauer spectra were collected in the temperature range 295–4 K in transmission mode using a conventional constant-acceleration spectrometer and a 25 mCi ^57^Co source in a Rh matrix. The velocity scale was calibrated using α-Fe foil. Isomer shifts, IS, are given relative to this standard at room temperature. The absorber was obtained by packing the powdered samples into a Perspex holder. The absorber thickness was calculated on the basis of the corresponding electronic mass-absorption coefficients for the 14.4 keV radiation. The low temperature spectra were collected in a bath cryostat with the sample immersed in liquid He at 4 K or in He exchange gas at 50 K. The spectra were fitted to Lorentzian lines using a non-linear least-squares method.

### Electrochemical measurements

The electrochemical experiments were performed using an Autolab electrochemical workstation (Autolab-128N potentiostat/galvanostat) connected to a personal computer that uses Nova 2.1 electrochemical software. The powdered materials were mixed with polytetrafluoroethylene (PTFE) in a mass ratio of 90 : 10 in ethanol and deposited on a 3 mm glassy carbon disc working electrode (which was polished sequentially with 0.3, 0.1 and 0.05 μm alumina powders and washed with deionised water before each experiment). A typical three-electrode experimental cell equipped with a platinum wire as the counter electrode and a silver wire as the pseudoreference electrode was used for the electrochemical characterization of the working electrodes. All measurements were carried out under magnetic agitation and nitrogen bubbling. The electrochemical properties were studied measuring the CV at different scan rates in 0.1 M TBAPF_6_/CH_3_CN solution. Ferrocene was added as an internal standard upon completion of each experiment. All potentials are reported in V *versus* Ag/AgCl.

### Catalytic experiments

5 mg of catalyst was placed into a round-bottom flask (25 mL). Activation of the **MUV-2** catalyst was carried out by heating at 150 °C under vacuum overnight. Subsequently, the reaction temperature was fixed at 140 °C and the required reaction atmosphere was obtained by purging the system with a balloon containing O_2_ under atmospheric pressure. The reaction time started by addition of a solution of DBT (200 mg L^–1^ of S) in 10 mL of the reaction solvent to the preheated round-bottom flask. Commercial diesel (Repsol) or *n*-dodecane were used as the reaction solvents. The mixture was continuously stirred magnetically at 500 rpm. The course of the reaction was followed by sampling 250 μL of the reaction mixture that was diluted with 250 μL of anisole and injected in a GC with a FI detector and a 30 m capillary column of 5% crosslinked phenyl methyl silicone. At the end of the reaction, the mixture was filtered to remove the solid while still hot and the organic phase was washed with three aliquots of 20 mL of water to remove the DBTO_2_ product formed in the process. Selective radical quenching experiments using *p*-benzoquinone or DMSO were carried out as described above but with the addition of 20 mol% of these reagents with respect to the initial DBT at 1.5 h of the reaction time.

## Author contributions

M. S. synthesised and characterised the material; G. M. E. and I. J. V.-Y. contributed to the solution and refinement of the structure from the single crystal data; A. S.-P., S. N. and H. G. carried out and analysed the catalytic experiments; B. J. C. V. and J. C. W. carried out and analysed the Mössbauer measurements; M. P., S. V. and F. R. carried out measurements and analysis of the adsorption isotherms; M. S. and G. M. E. conceived the research and prepared the manuscript; all authors made comments on the manuscript.

## Conflicts of interest

There are no conflicts to declare.

## Supplementary Material

Supplementary information

Crystal structure data
