# Supplementary material for: A highly stable and hierarchical tetrathiafulvalene-based metal–organic framework with improved performance as a solid catalyst
Source: Chem Sci. 2018 Jan 24;9(9):2413–8. doi: 10.1039/c7sc04829g (PMC5909329; doi:10.1039/c7sc04829g)
Supplement: Supplementary file 1 [file SC-009-C7SC04829G-s001.pdf]

Electronic Supplementary Material (ESI) for Chemical Science.
This journal is © The Royal Society of Chemistry 2018

Supporting Information

A Highly Stable and Hierarchical Tetrathiafulvalene-based Metal-Organic Framework with Improved Performance as Solid Catalyst

Manuel Souto,[a] Andrea Santiago-Portillo,[b] Miguel Palomino,[c] Iñigo J. Vitórica-Yrezábal,[d] Bruno J. C. Vieira,[e] João C. Waerenborgh,[e] Susana Valencia,[c] Sergio Navalón,[b] Fernando Rey,[b] Hermenegildo García,[b,c] Guillermo Mínguez Espallargas*[a]

[a] Instituto de Ciencia Molecular (ICMol), Universidad de Valencia, c/Catedrático José Beltrán, 2, 46980 Paterna, Spain

[b] Departamento de Química, Universitat Politècnica de València, C/Camino de Vera, s/n, 46022, Valencia, Spain

[c] Instituto de Tecnología Química (UPV-CSIC), Universitat Politècnica de València-Consejo Superior de Investigaciones Científicas, Av. De los Naranjos 46022 Valencia, Spain

[d] School of Chemistry, University of Manchester, Oxford Road, Manchester, M13 9PL, UK

[e] Centro de Ciências e Tecnologias Nucleares, Instituto Superior Técnico, Universidade de Lisboa, 2695-066 Bobadela, LRS, Portugal

Contents

1. **General methods and materials**
2. **Synthesis of Et_4TTFTB**
3. **Synthesis of H_4TTFTB**
4. **Synthesis of Fe_3O**
5. **Synthesis of MUV-2**
6. **IR of MUV-2**
7. **Crystallographic data**
8. **Mössbauer of MUV-2**
9. **SQUID of MUV-2**
10. **CV of MUV-2**
11. **Raman of MUV-2**
12. **EDAX of MUV-2**
13. **TGA of MUV-2**
14. **Chemical stability of MUV-2**
15. **Adsorption measurements of MUV-2**
16. **Catalytic activity of MUV-2**
17. **References**

1. General methods and materials

All reagents and solvents employed for the syntheses were of high purity grade and were purchased from Sigma-Aldrich Co., and TCI. ^{1}H NMR spectra were recorded using a Bruker DPX300 (300 MHz) spectrometer and Me_4Si as an internal standard. Infrared spectra were recorded in a FT-IR Nicolet 5700 spectrometer in the 4000-400 cm^{-1} range using powdered samples diluted in KBr pellets. Thermogravimetric analysis was carried out with a Mettler Toledo TGA/SDTA 851 apparatus in the 25-600 ºC temperature range under a 10 ºC/min scan rate and a N_2 flow of 30 mL·min^{-1}. Powder X-ray diffraction spectra were recorded using 0.7 mm borosilicate capillaries that were aligned on an Empyrean PANalytical powder diffractometer, using Cu Kα radiation (λ = 1.54056 Å).

2. Synthesis of Et_4TTFTB [1]

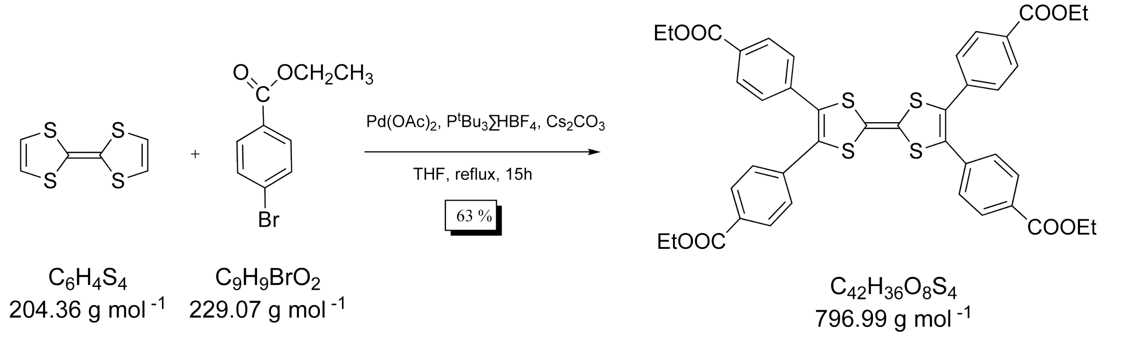

$Pd(OAc)_2$ (84 mg, 0.38 mmol), P^tBu_3•HBF_4 (326 mg, 1.1 mmol), and Cs_2CO_3 (2444 mg, 7.5 mmol) were placed in a 50-mL reaction flask under Argon. THF (10.0 mL) was added and the mixture was stirred for 10 min with heating. A solution of tetrathiafulvalene (307 mg, 1.5 mmol) and ethyl 4-bromobenzoate (1718 mg, 7.5 mmol) in THF (10.0 mL) was added. The mixture was heated at reflux for 15 h. The organic compounds were extracted with chloroform three times. The combined organic part was washed with brine, dried over anhydrous Na_2SO_4, and concentrated in vacuo. Chromatographic purification on silica gel by using hexane/dichloromethane as an eluent afforded 2,3,6,7-tetra(4-ethoxycarbonylphenyl) tetrathiafulvalene (Et_4TTFTB) (840 mg, 1.1 mmol, 70%) as a dark red solid. Characterization: ^{1}H NMR ($CDCl_3$): δ = 1.37 (t, J = 7.1 Hz, 12H), 4.36 (q, J = 7.1 Hz, 8H), 7.25–7.27 (m, 8H), 7.91 (dt, J = 8.7, 1.8 Hz, 8H). ^{13}C NMR ($CDCl_3$): δ = 14.51, 61.42, 108.92, 129.27, 130.06, 130.19, 130.80, 136.82, 166.02. IR (ν cm^{-1}): 1716 (s, C=O); 1696 (m); 1275 (s); 1101 (s).

3. Synthesis of H_4TTFTB [2]

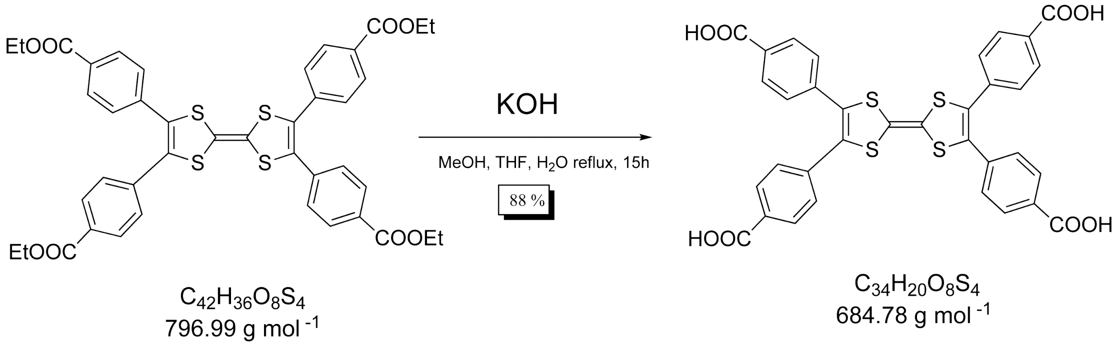

A 50-mL flask was charged with Et_4TTFTB (734 mg, 0.921 mmol) and subjected to three cycles of evacuation and refilling with Ar. Degassed MeOH (7 mL) and THF (7 mL) were added to generate a suspension. In a separate flask, NaOH (466 mg, 11.6 mmol) was dissolved in degassed water (5 mL). The sodium hydroxide solution was added to Et_4TTFTB under Ar and the reaction was heated to reflux for 12 hours. The reaction was then cooled to room temperature and the volatiles were removed in vacuo. A 1M solution of HCl (30 mL) was added to afford a maroon precipitate, which was collected by filtration and washed with water (50 mL). The product was collected and dried under high vacuum for 12 hours to afford H_4TTFTB as a maroon solid (556.5 mg, 0.813 mmol, 88% yield). ^{1}H NMR (DMSO-d_6): d = 13.14 (br s, 4H, CO_2H), 7.87 (dt, 8H, J = 8.3 Hz, 1.8 Hz, $CHCCO_2H$), 7.35 (dt, 8H, J = 8.4 Hz, 1.8 Hz, SCCCH). ^{13}C NMR (DMSO-d_6): d = 166.5, 135.5, 131.2, 129.9, 129.2, 129.1, 107.7. IR (ν cm^{-1}): 3420 (s, OH); 1686 (s, C=O); 1602 (s); 1412 (m); 1265 (m).

4. Synthesis of trinuclear Fe_3O [3,4]

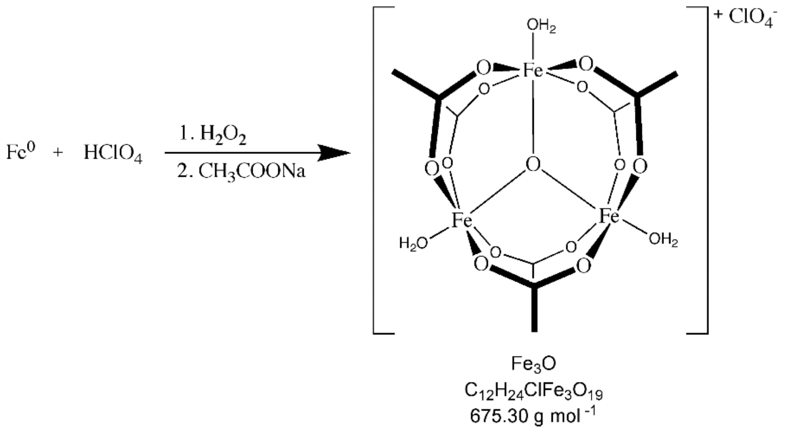

1.1 g of Fe^0 (0.02 mol) were dissolved in 10 ml of water (MQ) and 5.6 ml of $HClO_4$ (60 %) were added dropwise and the solution was stirred at 50-60ºC during 2 hours. The blue solution was cooled at room temperature and stirred overnight. Then 3.5 ml of H_2O_2 (35 %) and 0.6 ml of water were added dropwise to the solution with an ice bath and the solution change from blue to orange. Finally, 3.3 of CH_3COONa (0.04 mol) were carefully added at 0-5 ºC to the solution that became dark reddish. The solution was collected in a Petri dish and dark red crystals of $[Fe_3O(CH_3COO)_6]ClO_4$ appeared after slow evaporation at room temperature and were collected by filtration.

5. Synthesis of MUV-2

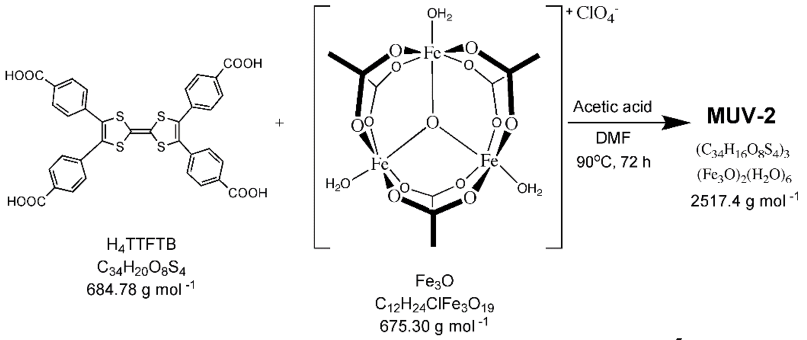

Single crystal: The procedure was adapted from ref. [5]. H_4TTFTB (5 mg), $[Fe_3O(CH_3COO)_6]ClO_4$ (5 mg) and acetic acid (0.2 ml) in 1 ml of DMF were ultrasonically dissolved in a 4 ml Pyrex vial. The mixture was heated in an oven at 90 ºC for 72 h. After cooling down to room temperature, dark red crystals were collected by filtration.

Bulk powder: 20 mg of H_4TTFTB, 20 mg of $[Fe_3O(CH_3COO)_6]ClO_4$ and 0.8 ml of acetic acid were dissolved in 4 ml of DMF in a 10 ml Pyrex vial. The mixture was heated in an oven at 90ºC for 72 h. After cooling down to room temperature, dark brown powder was collected by filtration. The powder was washed with large amount of DMF in order to remove the unreacted ligand and $[Fe_3O(CH_3COO)_6]ClO_4$ and immersed in DMF during one night. Finally, the product was immersed in EtOH during 3 h at 65ºC, washed and collected by filtration (19.6 mg; 80 %).

6. IR of MUV-2

The infrared spectrum of **MUV-2** clearly shows the presence of the vibrational bands characteristic of the framework -(O-C-O)- groups around 1550 and 1430 cm^{-1} confirming the presence of the dicarboxylate within the solid. On the other hand, the band at 1700 cm^{-1} characteristic of free C=O groups was not observed for washed **MUV-2** which is in agreement with the absence of free acid within the pores for this solid.[6]

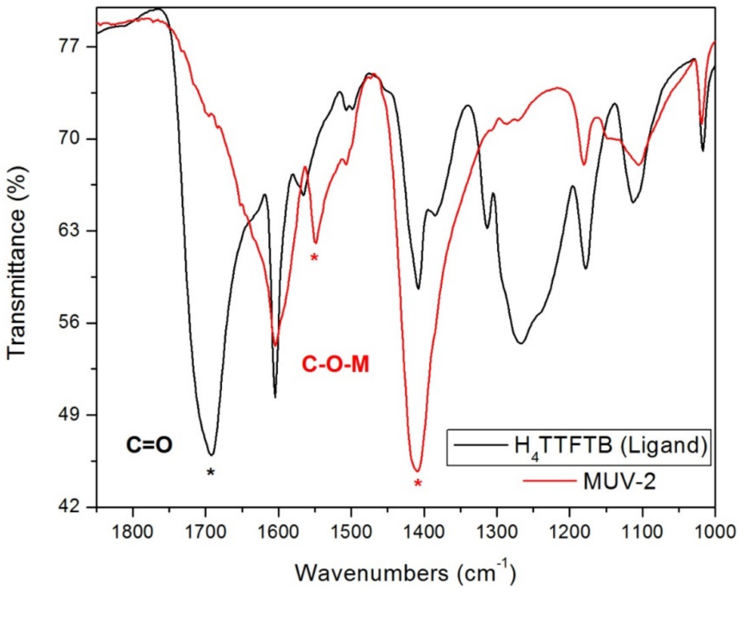

Fig. S1. IR of H_4TTFTB ligand and washed **MUV-2**.

7. Crystallographic data

Data Collection. X-ray data for compound **MUV-2** was collected at a temperature of 100 K using a synchrotron radiation at single crystal X-ray diffraction beamline I19 in Diamond light Source,[7] equipped with an Pilatus 2M detector and an Oxford Cryosystems nitrogen flow gas system. Data was measured using GDA suite of programs.

Crystal structure determinations and refinements. X-ray data were processed and reduced using CrysAlisPro suite of programmes. Despite of using Diamond Light Source synchrotron radiation, crystals of **MUV-2** only diffracted to 1.2 Å of resolution. Thus, the data was trimmed accordingly. Absorption correction was performed using empirical methods (SCALE3 ABSPACK) based upon symmetry-equivalent reflections combined with measurements at different azimuthal angles. The crystal was found to be an inversion twin with a twin law of (-1, 0, 0, 0, -1, 0, 0, 0, -1) and a BASF of 0.50(4). The crystal structure was solved and refined against all F^2 values using the SHELXL and Olex2 suite of programmes.[8,9] All atoms were refined anisotropically with the exception of the $[Fe_3O(O_2CCH_3)_6(H_2O)_3]^+$ cations. Hydrogen atoms were placed in the calculated positions. Starting material $[Fe_3O(O_2CCH_3)_6(H_2O)_3]^+$ clusters were found disordered and modelled over six positions in the pores. The C-O distances and O-C-C angles were restrained using SADI and DFIX commands. The atomic displacement parameters were also restrained using SIMU, DELU and RIGU commands. Solvent mask protocol in Olex2 was implemented to account with the remaining electron density corresponding to disordered molecules of starting material and solvent molecules. A total of 565 electrons were found un-accounted in the model. As Z =2, a total of 282 electrons per asymmetric unit of **MUV-2** were un-accounted, which could correspond to 0.5 molecule of ClO_4 and unknown number of un-coordinated ligand and solvent molecules. CCDC 1579606 contains the supplementary crystallographic data for this paper. These data can be obtained free of charge *via* www.ccdc.cam.ac.uk/conts/retrieving.html (or from the Cambridge Crystallographic Data Centre, 12 Union Road, Cambridge CB21EZ, UK; fax: (+44)1223-336-033; or deposit@ccdc.cam.ac.uk).

Table S1. Crystallographic information for **MUV-2**.

	**MUV-2**
Crystal colour	red
Crystal size (mm)	0.18 × 0.03 × 0.02
Crystal system	Hexagonal
Space group, Z	P-62m, 2
a (Å)	33.298(3)
c (Å)	12.3958(7)
V (Å^3)	11903(2)
Density (Mg.m^{-3})	0.429
Wavelength (Å)	0.6889
Temperature (K)	100
μ(Mo-Kα) (mm^{-1})	0.316
2Θ range (°)	3.622 to 33.358
Reflns collected	22307
Independent reflns (R_{int})	2697 (0.2068)
L.S. parameters, p	215
No. of restraints, r	199
$R1(F)^a$ $I > 2.0\sigma(I)$	0.0324
$wR2(F^2)$,[b] all data	0.0929
S (F^2)[c]	0.671

[a] $R1(F) = \Sigma(|F_o| - |F_c|)/\Sigma|F_o|$; [b] $wR^2(F^2) = [\Sigma w(F_o^2 - F_c^2)^2/\Sigma wF_o^4]^{½}$; [c] $S(F^2) = [\Sigma w(F_o^2 - F_c^2)^2/(n + r - p)]^{½}$

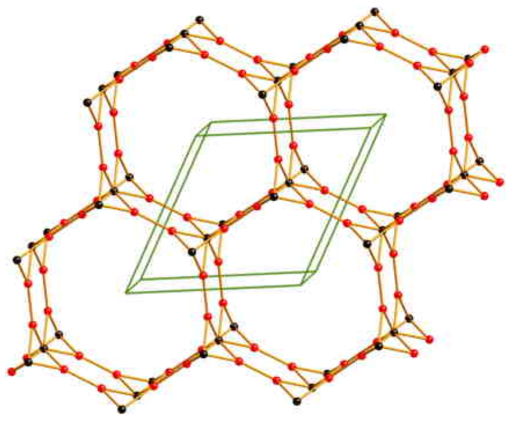

Fig. S2. Schematic representation of the 4,6-connected network with **ttp** topology

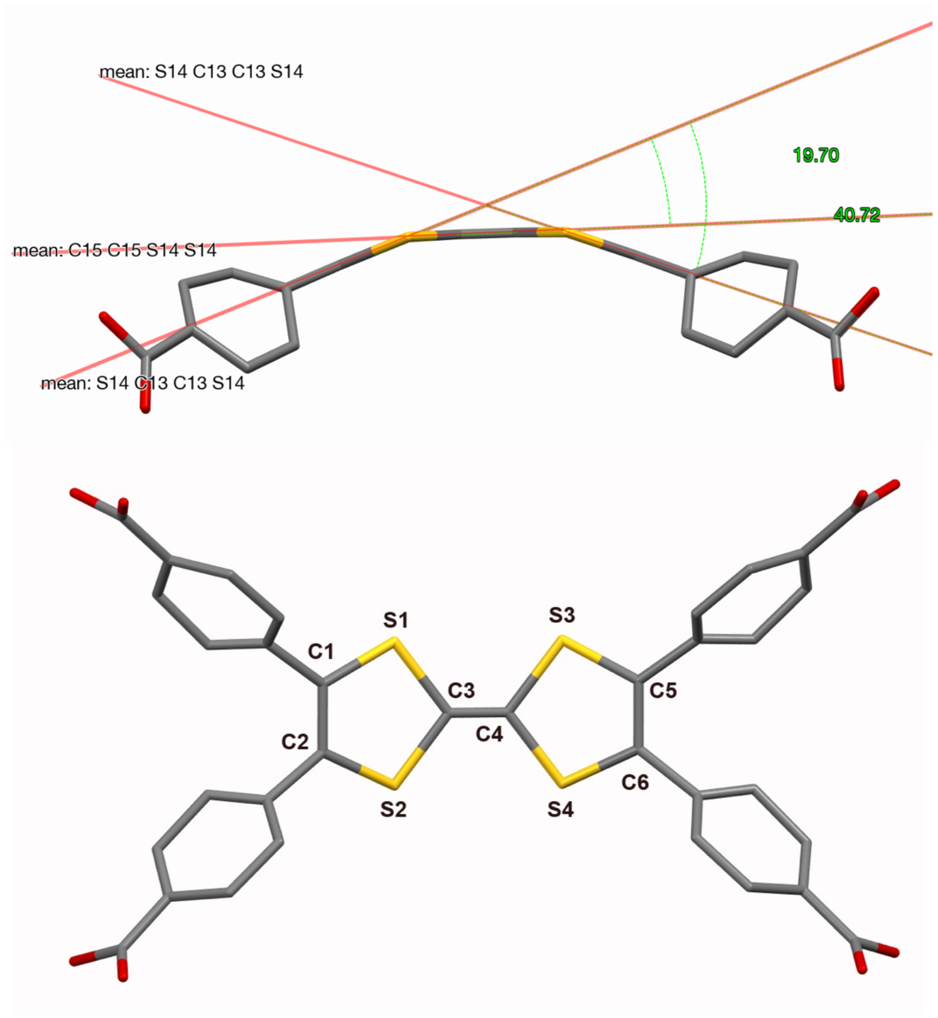

Fig. S3. Partial views of the crystal structure of TTFTB in **MUV-2** highlighting the dihedral angles.

8. Mössbauer of MUV-2

Mössbauer spectra were collected in the temperature range 295–4 K in transmission mode using a conventional constant-acceleration spectrometer and a 25 mCi ^{57}Co source in a Rh matrix. The velocity scale was calibrated using α-Fe foil. Isomer shifts, IS, are given relative to this standard at room temperature. The absorber was obtained by packing the powdered samples into a Perspex holder. The absorber thickness was calculated on the basis of the corresponding electronic mass-absorption coefficients for the 14.4 keV radiation.[10] The low temperature spectra were collected in a bath cryostat with the sample immersed in liquid He at 4 K or in He exchange gas at 50 K. The spectra were fitted to Lorentzian lines using a non-linear least-squares method.[11]

The Mössbauer spectra (Fig. S4) of **MUV-2** in the temperature range 4-295 K consist of two absorption peaks. At 295 and 50 K they may be fitted by a single, symmetric, quadrupole doublet. The estimated isomer shifts relative to metallic Fe at room temperature, IS, are consistent with S=5/2 Fe(III) ion in an octahedral oxygen environment[12] and similar to those reported for other Fe(III) trinuclear complexes.[13–16] Considering that S=5/2 Fe(III) has significantly lower quadrupole splittings than S=2 Fe(II),[12] if Fe in this electronic state was present it would give rise to one resolved peak located at a higher velocity than the S=5/2 Fe(III) peaks. The room temperature spectrum was accumulated during a long time in order to improve the statistics, so the dispersion of the base line counts is narrow enough to allow the detection of this S=2 Fe(II) peak if it had > 1% of the total absorption area. Since this peak was not observed, if there is any S=2 Fe(II) in the sample its content should be ≤ 2% of the total Fe. The occurrence of a single doublet is in fair agreement with the similarity of the first coordination spheres of the Fe(III) cations in the three different crystallographic sites. The increase in IS with decreasing temperature is due to the second order Doppler shift.

The line widths, Γ, at 4 K are significantly larger than at room temperature and 50 K. Furthermore, the doublet is clearly asymmetric. These features may be explained by a decrease of the relaxation frequency of the direction of the Fe(III) magnetic moments due to the presence of strong AFM interactions (see below magnetic measurements).[12,13,15,16] The relaxation frequency, however, is not low enough to allow the observation of magnetic splitting, which implies that no long range magnetic correlations are established.

In order to maintain the charge balance, one of the 3 coordinated H_2O molecules in the cluster [$Fe_3(\mu_3\text{-}O)$] is present as a negatively charged hydroxide OH^- and the formula for a neutral **MUV-2** is $(C_{34}H_{16}O_8S_4)_3(Fe_3O)_2(H_2O)_4(OH)_2$.

Table S2. Estimated parameters from the Mössbauer spectra of MOF based on Fe_3O trimers sample taken at different temperatures.

T	IS (mm/s)[a]	QS (mm/s)[b]	Γ (mm/s)[c]
295 K	0.41	0.71	0.54
50 K	0.54	0.73	0.68
4 K	0.54	0.82	0.98

[a] IS (mm/s) isomer shift relative to metallic α-Fe at 295 K; [b] QS (mm/s) quadrupole splitting; [c] Γ peak width. Estimated errors are ≤ 0.02 mm s^{-1} for IS and QS, Γ.

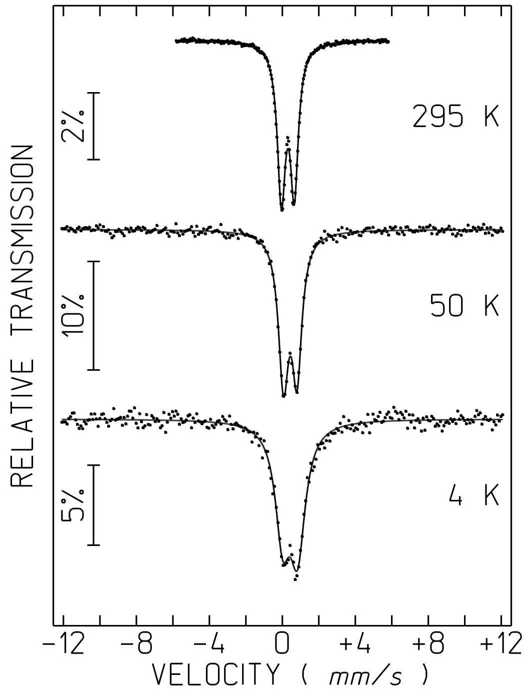

Fig. S4. Mössbauer spectra of **MUV-2** taken at different temperatures.

9. SQUID of MUV-2

The temperature dependence of the magnetic susceptibility (Figure S5) shows that χT product of **MUV-2** at 300 K (11.9 cm^3·K·mol^{-1}) is closed to the theoretical expected value for three Fe(III) non-interacting S = 5/2 spins (χT = 13.125 cm^3·K·mol^{-1}). Upon cooling, χT strongly decreases when lowering the temperature in agreement with the strong antiferromagnetic coupling within the quasi-isolated Fe(III) trimers.

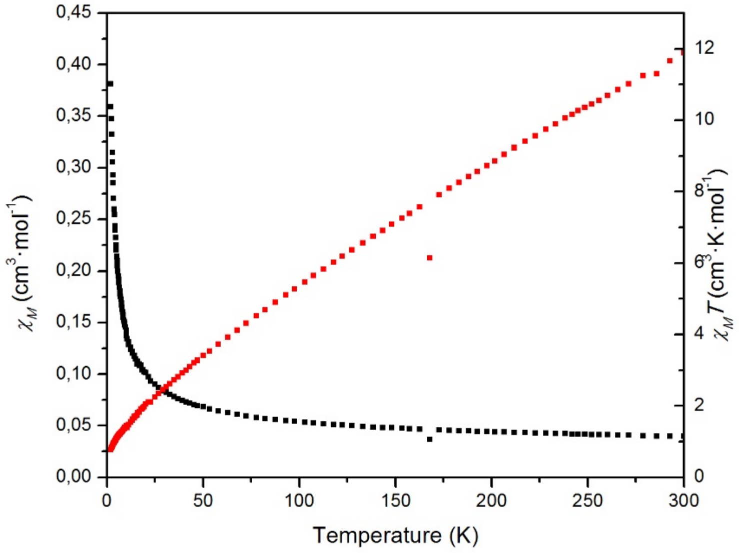

Fig. S5. Magnetic susceptibility χ (black line) and χT (red line) versus temperature variation of **MUV-2**.

10. CV of MUV-2

Solid-state cyclic voltammetry (CV) of **MUV-2** (Figure S6) was recorded in the potential range of –1.2 to 1.6 V and exhibited one reversible reduction peak at –0.77 corresponding to the Fe^{3+}/Fe^{2+} reduction. Surprisingly, it was not observed any peak corresponding to the oxidation process of the electroactive TTF ligand. As it has been previously reported for other bent TTF derivatives,[17,18] the significant deviation from planarity of the TTF unit destroys its donor ability by preventing efficient 6-π electron delocalization (heteroaromaticity) within the dithiolium cation formed in the $TTF^{+\cdot}$ radical cation. Moreover, the oxidation of TTF ligand of **MUV-2** with iodine was not successful as confirmed by Raman spectroscopy (see below).

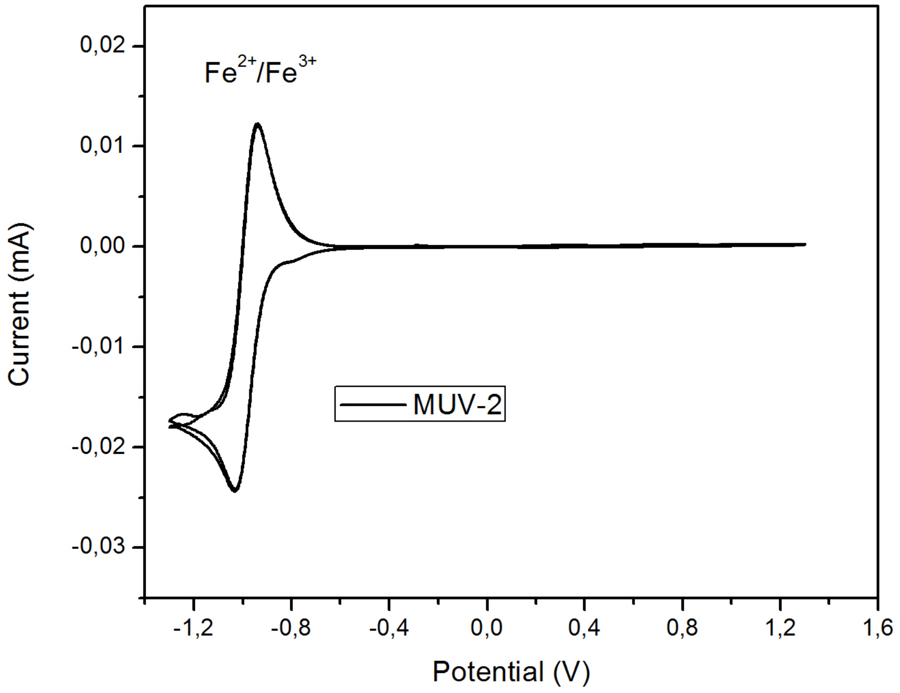

Fig. S6. Solid-state cyclic voltammetry of **MUV-2** collected at 0.05 V/s in $TBAPF_6/CH_3CN$ 0.1 M electrolyte using a platinum wire as the counter electrode, and a silver wire as the pseudoreference electrode. Ferrocene was added as an internal standard upon completion of each experiment. All potentials are reported in V versus Ag/AgCl.

11. Raman of MUV-2

Raman spectrum of **MUV-2** shows that the peak assigned to C=C stretch of the neutral TTF core which appears at 1605 cm^{-1} for the ligand slightly shift to 1609 cm^{-1} for **MUV-2** confirming that the TTF remains in the neutral state (Figure S7).[19] Moreover, it was intended to chemically oxidize the TTF ligands of **MUV-2** via iodine doping by immersing the crystals in a solution of iodine in cyclohexane as it has been reported for other TTF-based MOF.[19] However, Raman peaks assigned to the C=C ring stretch of the TTF moiety in **MUV-2@I$_2$** remained identical and didn't shift towards lower frequencies confirming that it was not possible to oxidize the TTF units with iodine (Figure S8).

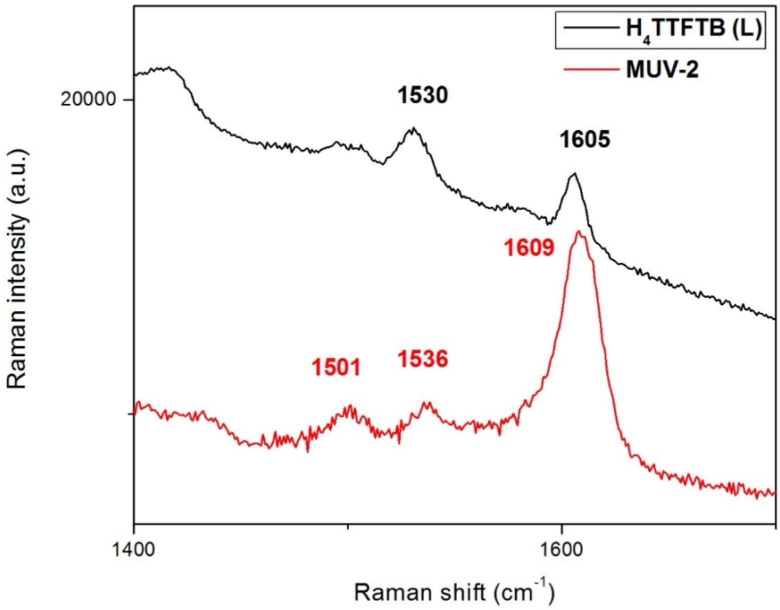

Fig. S7. Solid state Raman spectra of the ligand **H_4TTFTB** and **MUV-2** using a laser of λ = 785 nm.

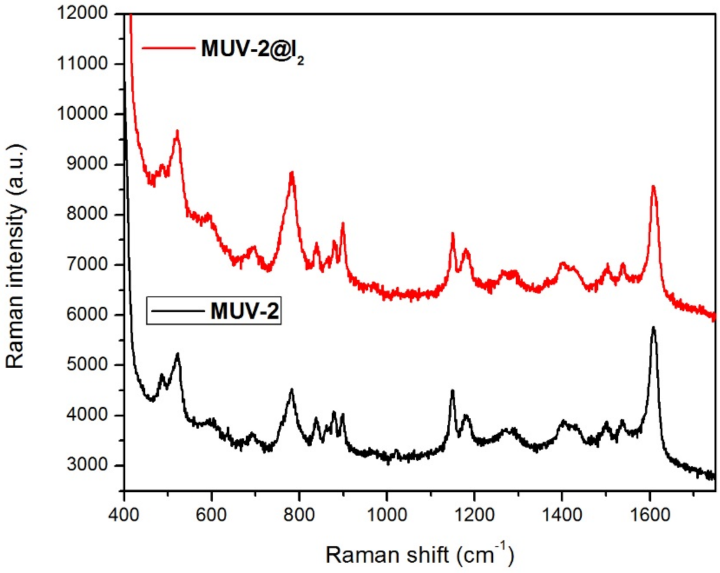

Fig. S8. Solid state Raman spectra of **MUV-2** and **MUV-2@I$_2$** using a laser of λ = 785 nm.

12. EDAX of MUV-2

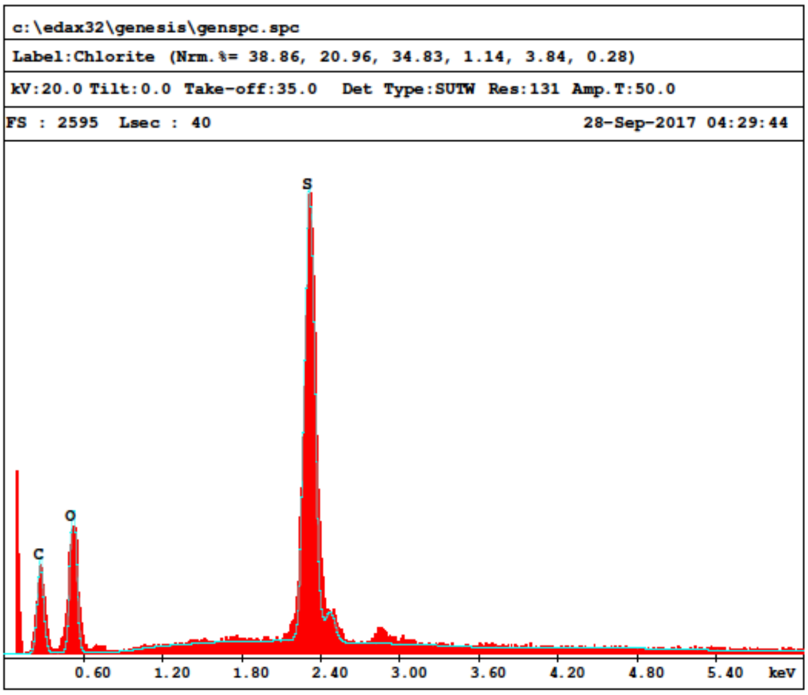

EDAX ZAF Quantification (Standardless)
Element Normalized
SEC Table : Default

Element	Wt %	At %	K-Ratio	Z	A	F
C K	47.26	63.93	0.0939	1.0336	0.1923	1.0002
O K	23.89	24.26	0.0469	1.0163	0.1929	1.0004
S K	15.83	8.02	0.1412	0.9575	0.9303	1.0012
FeK	13.03	3.79	0.1130	0.8582	1.0108	1.0000
Total	100.00	100.00				

Element	Net Inte.	Bkgd Inte.	Inte. Error	P/B
C K	57.63	0.90	2.12	64.03
O K	89.13	1.20	1.70	74.27
S K	454.00	16.05	0.77	28.29
FeK	138.93	5.63	1.39	24.70

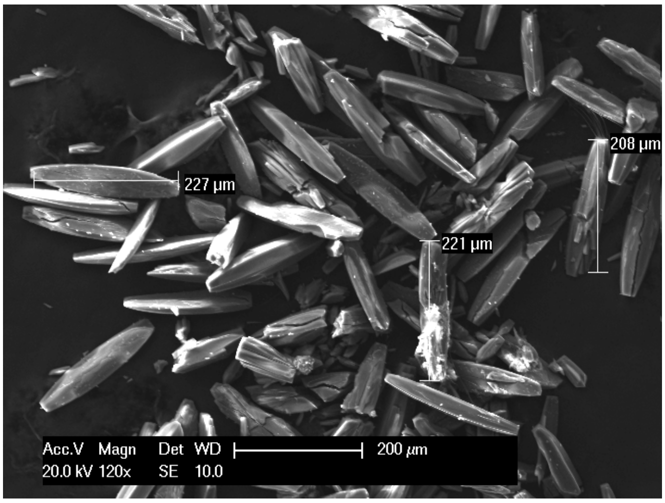

Fig. S9. Top: EDAX analysis of washed crystals of washed **MUV-2** confirming the presence of all the elements (C, O, S, Fe) and the (S : Fe) ratio of (2 : 1). Bottom: Picture of SEM showing the length of the crystals (100-200 μm).

13. TGA of MUV-2

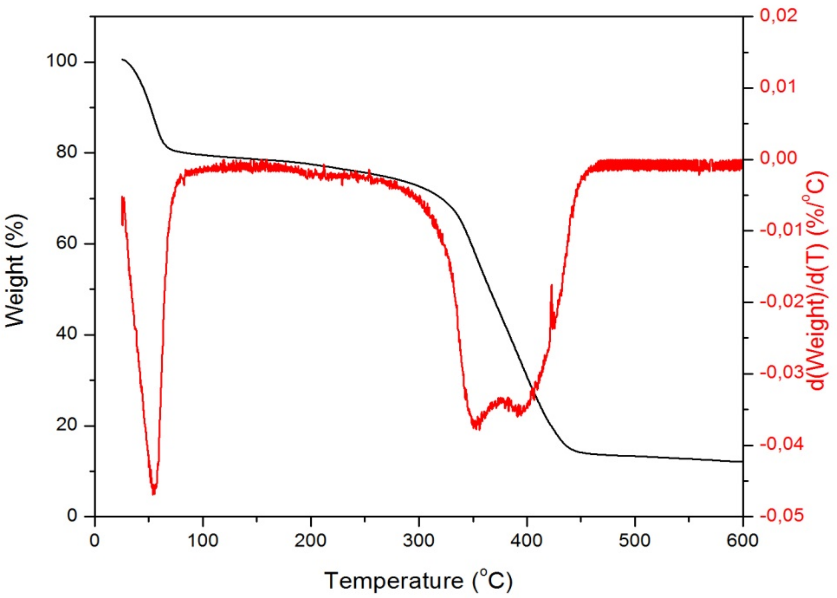

Fig. S10. Thermogravimetric analysis (TGA) profile of washed **MUV-2** at a heating rate of 5ºC/min under a constant stream of N_2 (black) and DTG curve (red).

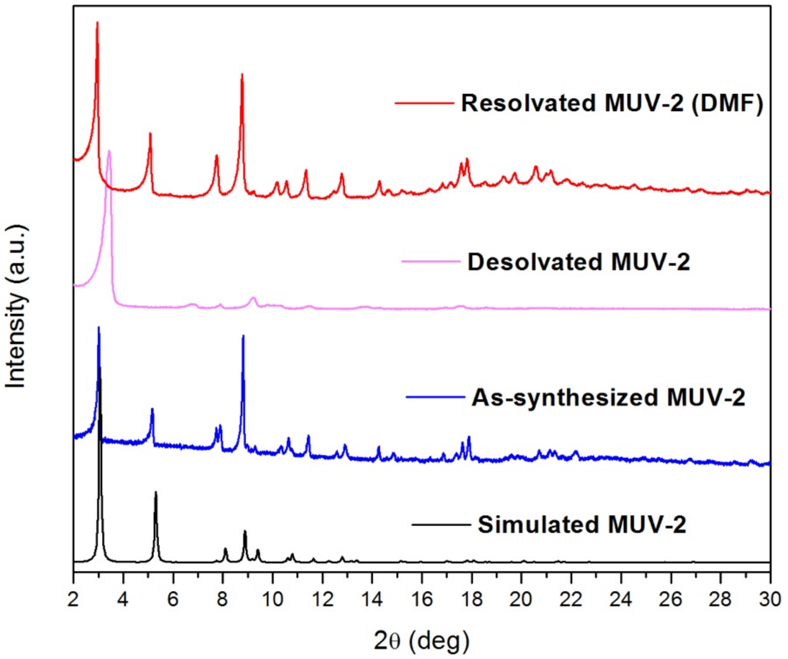

Fig. S11. Powder X-ray diffraction patterns of simulated, as-synthesised, desolvated and resolvated (DMF) of **MUV-2**.

14. Chemical stability of MUV-2

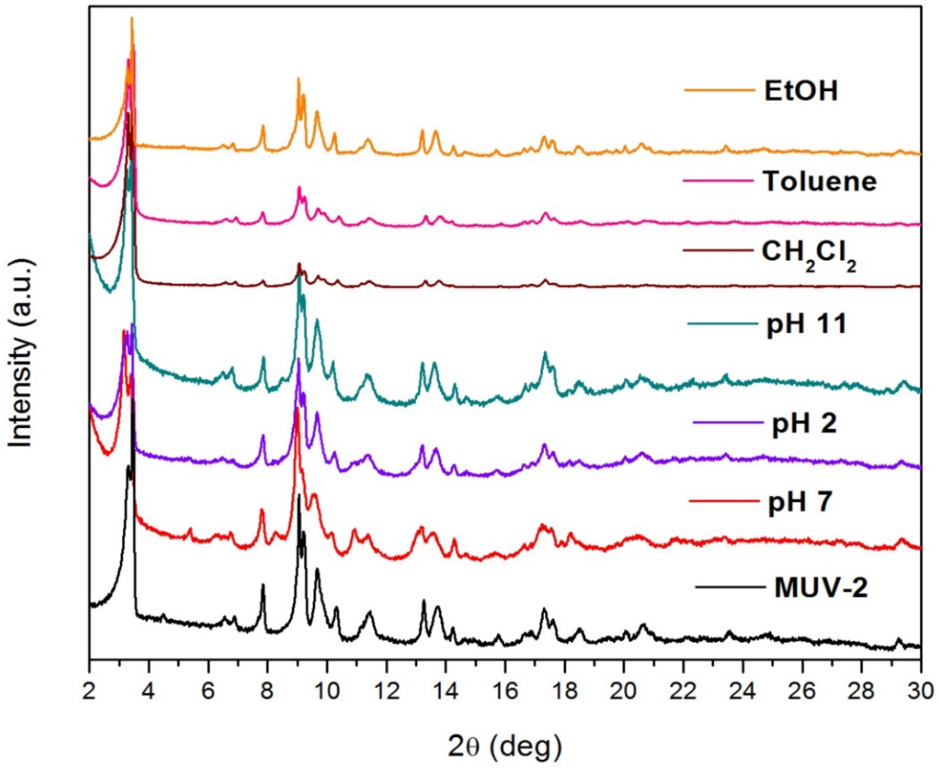

Fig. S12. Powder X-ray diffraction patterns of washed **MUV-2** and after treatment with aqueous solutions of pH = 2, 7 and 11, and with different organic solvents (CH_2Cl_2, toluene, EtOH) for 24 h. Concentration: 5 mg of **MUV-2** / 5 ml buffer solution.

15. Adsorption measurements of MUV-2

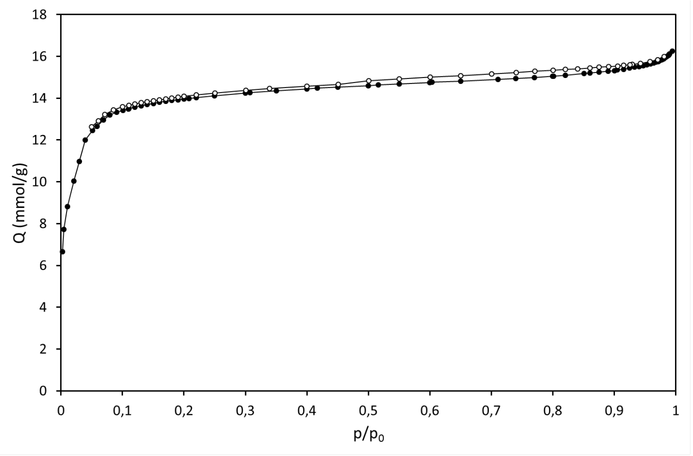

Fig. S13. Nitrogen adsorption isotherm at 77 K on **MUV-2**.

The BET surface area was determined applying the theory of Brunauer, Emmett, and Teller,[20] but fulfilling the criterion established by Rouquerol *et al.*[21] (Figure S11), according to which the relative pressure range for BET surface area determination was limited to 0-0.08.

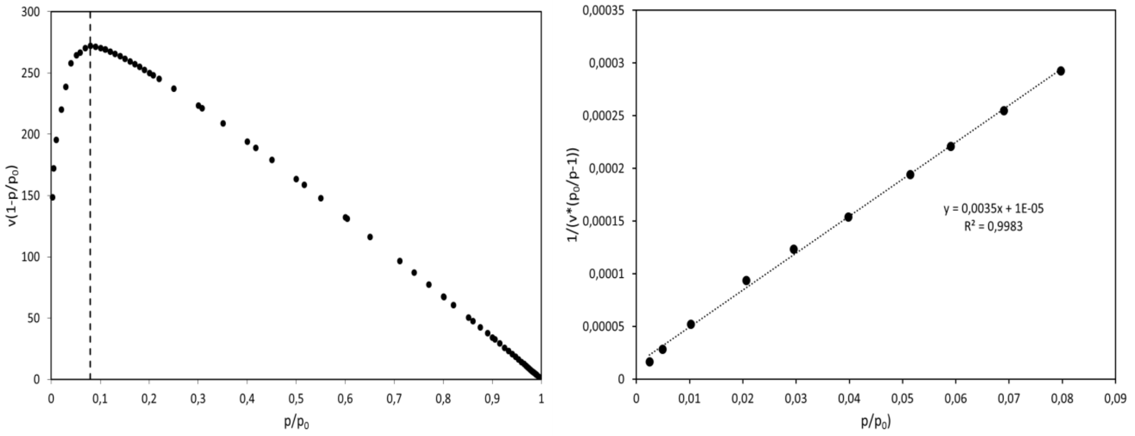

Fig. S14. $v(1-p/p_0)$ vs p/p_0 (left) and BET plot for the 0-0.08 relative pressure range (right).

The pore size distribution was obtained by means of the Barrett-Joyner-Halenda (BJH) method and the Harkins-Jura statistical film thickness curve (t-curve), shown in Figure S15. The average pore diameter was 38.7 Å.

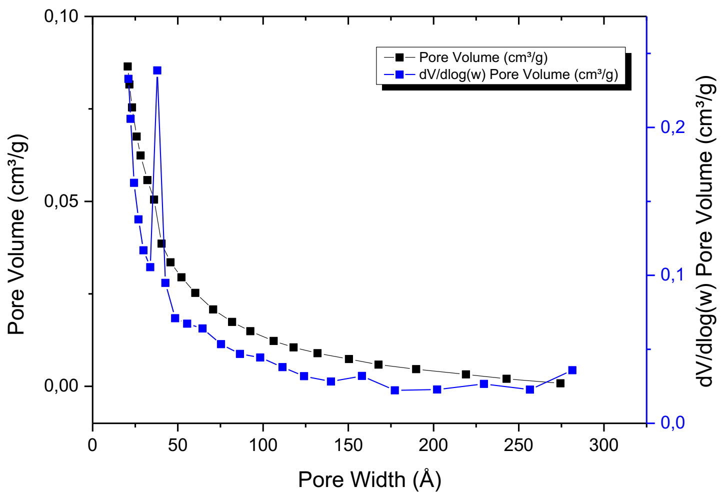

Fig. S15. BJH pore size distributions for **MUV-2**.

The surface area of **MUV-2** was also calculated through the monolayer capacity of CO_2 at 298 K by using Dubinin-Astakhov equation.[22] In this case, it was assumed that the molecular sectional area of CO_2 at 298 K is 0.195 nm^2.[23] The surface area of **MUV-2** calculated via the CO_2 Dubinin-Astakhov approach is 1425 m^2/g at 298 K (Figure S16). This value is in good agreement with the corresponding obtained with the N_2 isotherm at 77 K and the BET model.

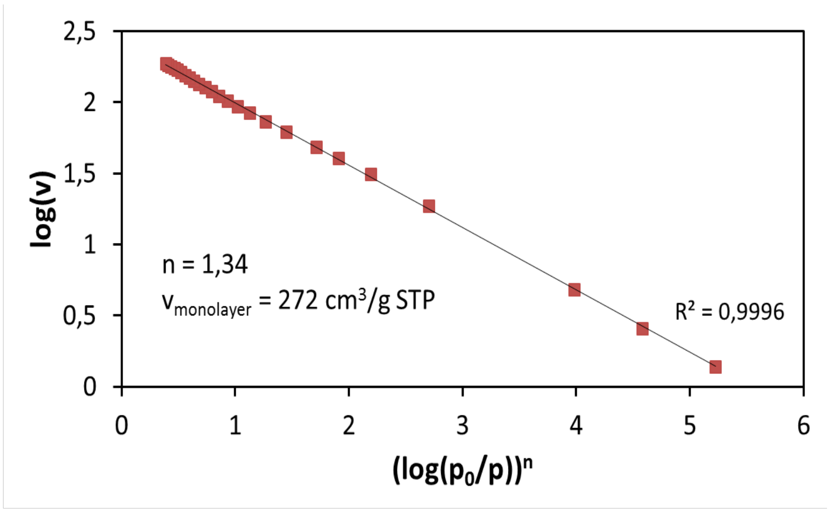

Fig. S16. Dubinin-Astakhov plot for CO_2 adsorption at 298 K on **MUV-2**.

The experimental isotherms were fitted by using a fourth grade virial equation: [24]

$$\ln\left(\frac{P}{Q}\right) = A_0 + A_1 Q + A_2 Q^2 + A_3 Q^3 + A_4 Q^4$$

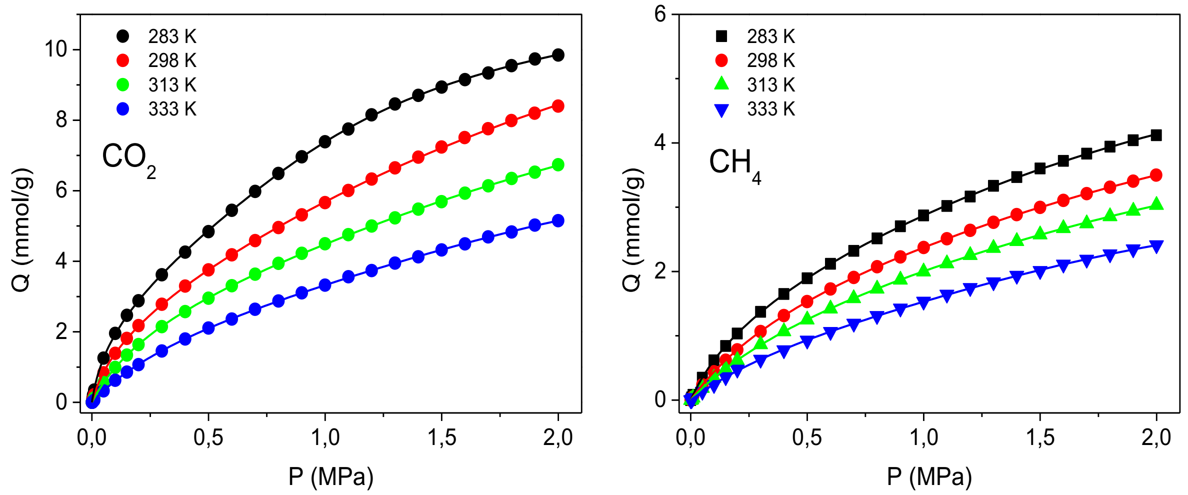

Fig. S17. Gas adsorption isotherms of CO_2 (left) and CH_4 (right) on **MUV-2** at different temperatures. Points are experimental uptakes and lines are virial fittings.

The isosteric heats of adsorption of CO_2 and CH_4 were calculated from sets of fitted isotherms according to the Clausius-Clapeyron equation: [25]

$$q_{st} = R \cdot T^2 \cdot \left[\frac{\partial(\ln P)}{\partial T}\right]_{Q=cte} \equiv -R \cdot \left[\frac{\partial(\ln P)}{\partial\left(1/T\right)}\right]_{Q=cte}$$

The isosteric heat of adsorption (q_{st}) of CO_2 decreases from 30 to 20 kJ/mol, which remains constant in the case of CH_4 at around 16 kJ/mol within the studied loading range (Figure S18). These values clearly indicate the highest affinity of **MUV-2** for the CO_2, rather than for the

CH_4. The isosteric heat of adsorption of CO_2 at zero coverage (q_{st}^0) of **MUV-2** is comparable to that of a LTA zeolite of Si/Al ratio of around 6.[26]

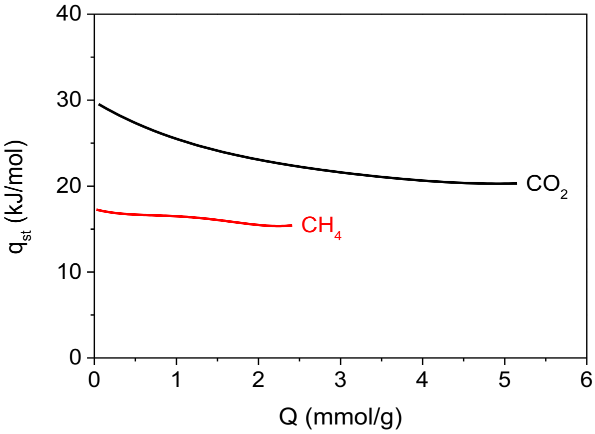

Fig. S18. Isosteric heat of adsorption of CO_2 and CH_4 on **MUV-2**.

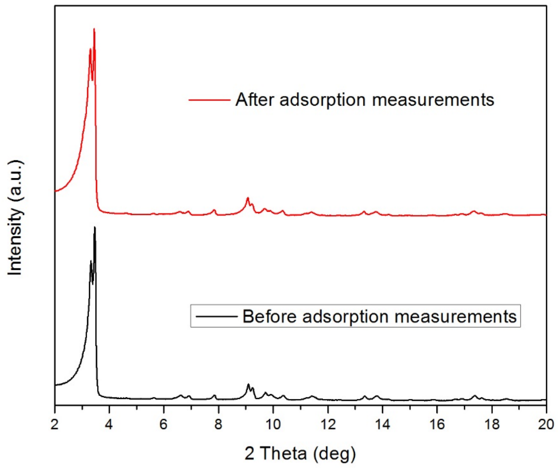

Fig. S19. PXRD of activated **MUV-2** before and after adsorption measurements.

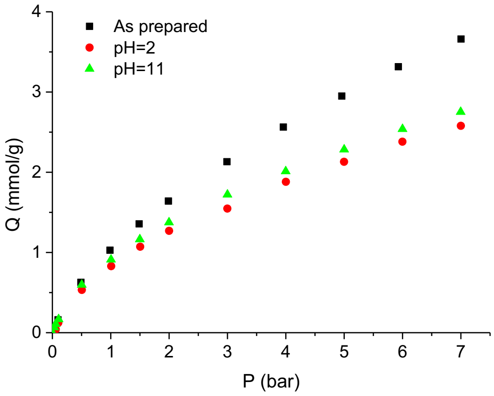

Fig. S20. Gas adsorption isotherms of CO_2 on **MUV-2** after treatment with pH = 2 and 11 aqueous solution.

16. Catalytic activity of MUV-2

Materials

DBT (99%), $DBTO_2$ (97%), 4-methyl DBT (96%) and 4,6-dimethyl DBT (97%) and *n*-dodecane were of analytical grade and supplied by Sigma-Aldrich.

Catalytic experiments

5 mg of catalyst was placed on a round-bottom flask (25 mL). Activation of the **MUV-2** catalyst was carried out by heating at 150 °C under vacuum overnight. Subsequently, the reaction temperature was fixed at 140 °C and the required reaction atmosphere was obtained by purging the system with a balloon containing O_2 under atmospheric pressure. The reaction time started by addition of a solution of DBT (200 mg L^{-1} of S) in 10 mL of the reaction solvent to the preheated round-bottom flask. As reaction solvent, *n*-dodecane or commercial diesel (Repsol) were used. The mixture was continuously stirred magnetically at 500 rpm. The course of the reaction was followed by sampling 250 µl of the reaction mixture that was diluted with 250 µl of anisole and injected in a GC having a FI detector and a 30 m capillary column of 5% crosslinked phenyl methyl silicone. At the end of the reaction, the mixture was filtered to remove the solid while still hot and the organic phase was washed with three aliquots of 20 ml of water to remove the $DBTO_2$ product formed in the process.
Selective radical quenching experiments using *p*-benzoquinone or DMSO were carried out as described above but with the addition of 20 mol% of these reagents with respect to the initial DBT at 1.5 h of the reaction time.

Raman measurements

Raman spectra were recorded with a 514 nm laser excitation on a Renishaw Raman Spectrometer ("Refelx") equipped with a CCD detector. The laser power on the sample was 25 mW and a total of 50 acquisitions were taken for each spectrum. A commercial Linkam cell (THMS600) was used for collecting spectra under controlled conditions. Thus, the sample was first subjected to a Ar flow (20 ml min^{-1}) at 220 °C for ~5 h.

Afterwards the temperature was lowered to 140 ºC in Ar, and then the gas was changed to O_2 (20 ml min^{-1}) at the same temperature. Finally, the gas was changed to Ar.

Table S3. Comparison of pore sizes and pore windows between the different MOFs **MIL-100**, **MIL-101** and **MUV-2**.

	**MIL-100**	**MIL-101**	**MUV-2**
**Pore window**	Pentagonal: 4.7 x 5.5 Å<br>Hexagonal: 8.6 x 8.6 Å	Pentagonal:12 x 12 Å<br>Hexagonal: 16 x 15 Å	Hexagonal: 33 x 33 Å<br>Trigonal: 12 x 9.5 Å
**Pore size**	25 - 29 Å	29 - 34 Å	33 Å

16.1. Study of the induction period

To gain understanding on the origin of the induction period, providing evidence of the favorable role of **MUV-2** mesoporosity explaining its higher catalytic activity, three control experiments were carried out in where **MUV-2** was contacted with *n*-dodecane containing DBT in the absence of oxygen for 2 h and then oxygen was admitted or **MUV-2** was boiled at the reaction temperature in *n*-dodecane under oxygen and then DBT was added after 2 h. In a third experiment, **MUV-2** was heated in *n*-dodecane under Ar for 2 h and then O_2 and DBT were added simultaneously. The results obtained (see Figure S21) clearly indicate that the induction period is significantly reduced to about one half when either DBT or O_2 are already present in the system and it is unchanged when both, DBT and O_2 are introduced in the system even if **MUV-2** was already equilibrated in *n*-dodecane. Therefore, these variations in the induction period indicate that diffusion of DBT and O_2 are responsible for the delay in observing DBT consumption or $DBTO_2$ appearance. Thus, the large pore size of **MUV-2** should be responsible for the shortened induction period observed for this hierarchical MOF respect to MIL-100 and MIL-100.

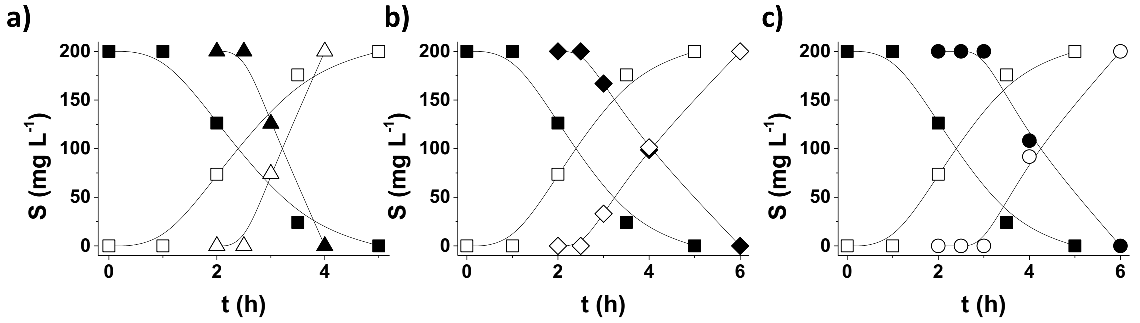

Fig. S21. Influence on the aerobic oxidation of DBT (▲) to $DBTO_2$ (Δ) using **MUV-2** as a catalyst at various pretreatments in comparison to the general procedure of the aerobic oxidation of DBT (■) to $DBTO_2$ (□). (a) DBT in *n*-dodecane with **MUV-2** under Ar at 140 °C for 2 h and then sudden addition of O_2 at 2 h; (b) suspending **MUV-2** in *n*-dodecane under O_2 at 140 °C for 2 h and then addition of DBT at 2 h; (c) contacting **MUV-2** in *n*-dodecane under Ar for 2 h at 140 °C and then sudden addition of DBT under an O_2 atmosphere at 2 h. Reaction conditions: catalyst (5 mg of **MUV-2**), DBT (1150 mg L^{-1}), *n*-dodecane (10 mL), O_2 (1 atm), 140 ºC. Note that for ▲(a), ◆ (b) and ● (c), the initial reaction time was 2 h.

16.2. Reusability of MUV-2

Stability of **MUV-2** as catalyst was addressed by performing a series of consecutive uses of the same sample recovered after each reaction, washed with anisole and then acetonitrile, dried and employed in a subsequent run under the general reaction conditions. The results comparing the time-conversion plot show that the induction period remains constant upon reuse, while, the reaction rate exhibits some decrease from the first to the third use (from 25.7 to 21.7 mgS h^{-1}) but it remains constant up to the fifth reuse (see Figure S22).

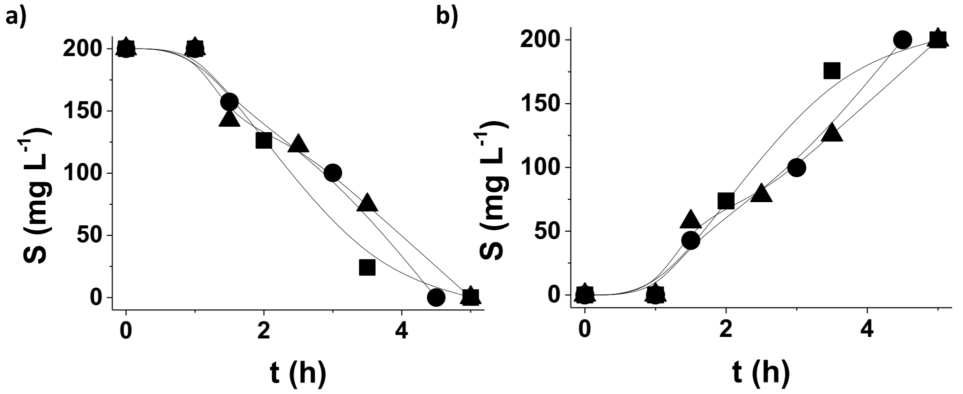

Fig. S22. Reusability of **MUV-2** as catalyst in the aerobic oxidation of DBT (a) to $DBTO_2$ (b). First use (■), third use (●) and fifth use (▲). Reaction conditions: catalyst (5 mg of **MUV-2**), DBT (1150 mg L^{-1}), *n*-dodecane (10 mL), O_2 (1 atm), 140 ºC.

16.3. Productivity test

MUV-2 stability of catalyst was also confirmed by performing a productivity test in where a large excess of DBT (1 g of S L^{-1}) was submitted to oxidation using a minor weight of catalyst (2 mg), allowing the reaction to progress for sufficiently long time. It was observed that a conversion about 95% of DBT can be achieved at 48 h reaction time even under these highly unfavourable conditions. (see Figure S23).

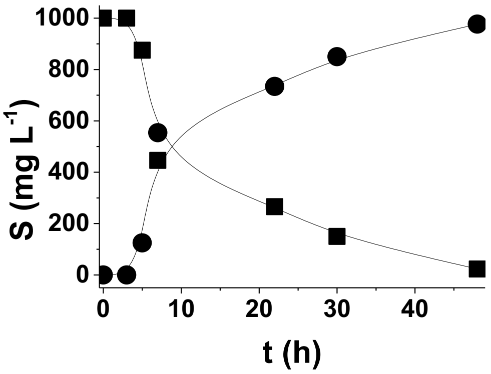

Fig. S23. Productivity test for the aerobic oxidation of DBT (■) to the corresponding sulfone (●) using **MUV-2** as catalyst. Reaction conditions: catalyst (2 mg of **MUV-2**), DBT (5750 mg L^{-1}), *n*-dodecane (10 mL), O_2 (1 atm), 140 ºC.

16.4. Scope of the reaction

The general scope of **MUV-2** as catalyst for the aerobic oxidation of DBTs was also evaluated by carrying out the oxidation of two derivatives containing one or two methyl groups (4-MeDBT and 4,6-Me_2DBT). As expected also for 4-MeDBT and 4,6-Me_2DBT oxidation to the corresponding sulfones proceeds in the presence of **MUV-2** as catalyst, although a gradual increase in the induction period was observed due to the larger molecular dimensions of these derivatives, following the order DBT<4MeDBT<4,6Me2DBT (see Figure S24).

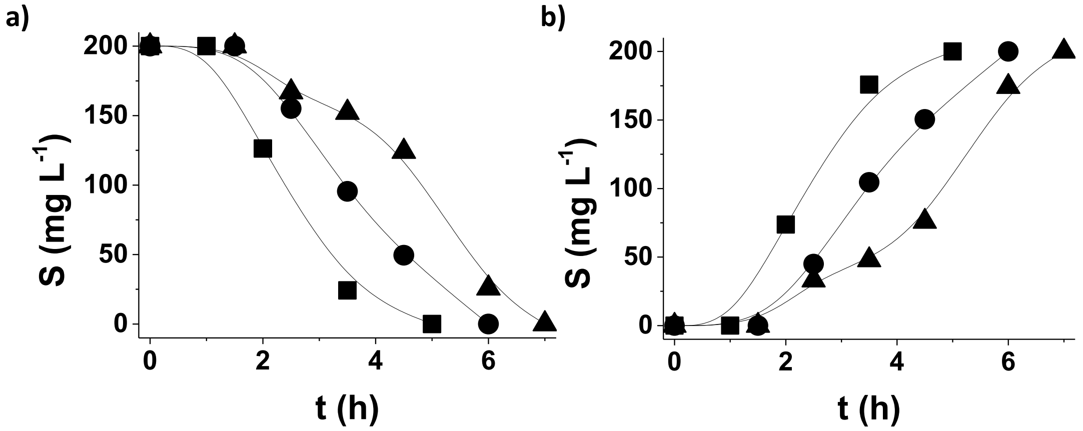

Fig. S24. (a) Aerobic oxidation of (■) DBT, (●) 4-MeDBT and (▲) 4,6-Me_2DBT to (b) the corresponding sulfones. Reaction conditions: catalyst (5 mg of **MUV-2**), DBT, 4-MeDBT and 4,6-Me_2DBT (1150 mg L^{-1}), *n*-dodecane (10 mL), O_2 (1 atm), 140 ºC.

16.5. Homogeneous, ligand and hot filtration test of MUV-2

Aerobic oxidation of DBT to $DBTO_2$ was also tested with the preformed cluster $[Fe_3O(CH_3COO)_6]ClO_4$ as homogeneous catalyst (Figure S25a,●) using the same iron amount as that employed when using **MUV-2** as heterogeneous catalyst (a,) or with the iron amount determined during the leaching of **MUV-2** (Figure S50a, □). Figure S50b show a blank control experiment where the ligand H_4TTFTB has been employed as homogeneous catalyst. Hot filtration test comparing the time-conversion plot of twin reactions in which for one of them the solid catalyst is removed by filtration at reaction temperature (140 ºC), when conversion was about 40%, shows that the process is mainly heterogeneous (about 85%), taking place in the solid catalyst (see Figure S25c).

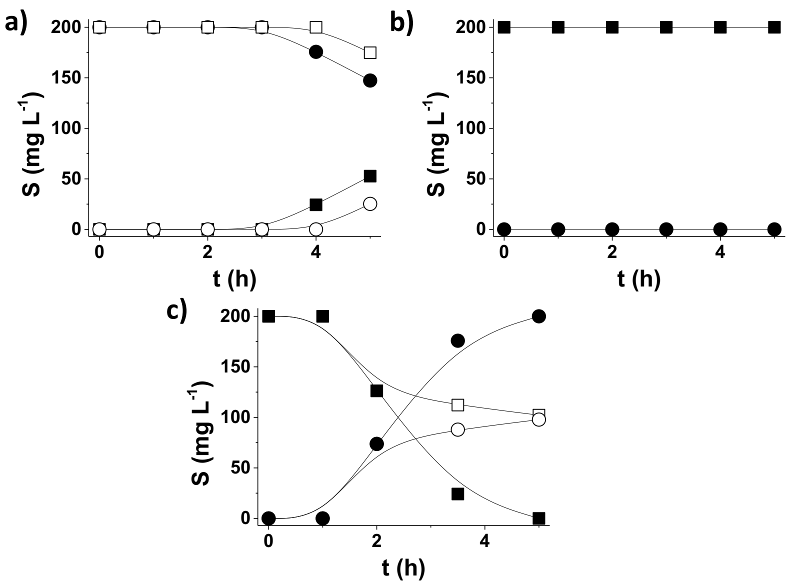

Fig. S25. a) Aerobic oxidation of DBT to DBTO2 using the preformed cluster $[Fe_3O(CH_3COO)_6]ClO_4$ as catalyst with the amount of Fe present in **MUV-2** (0.014mmol) DBT (■), $DBTO_2$ (●); with the amount of Fe leached (0.00007mmol) DBT (□), $DBTO_2$ (○), **b)** Aerobic oxidation of DBT to $DBTO_2$ using the ligand H_4TTFTB as catalyst with the amount of ligand present in MUV-2 (4 mg) DBT (■), $DBTO_2$ (●), **c)** Hot filtration test for the aerobic oxidation of DBT to $DBTO_2$ using **MUV-2** as catalyst. In the presence of catalyst DBT (■), $DBTO_2$ (●); after catalyst filtration DBT (□), $DBTO_2$ (○) at 40 % conversion. Reaction conditions: catalyst (5mg of **MUV-2**), DBT (1150 mg L^{-1}), *n*-dodecane (10 mL), O_2 (1 atm), 140 ºC.

16.6. Quenching experiments

To address the reaction mechanism and, particularly, to determine the nature of the reactive oxygen species responsible for oxidation, a combination of quenching experiments and spectroscopic studies were carried out. Thus, while DMSO (a specific hydroxyl radical quencher) exerts a minor influence on the temporal profile of the oxidation, addition of *p*-benzoquinone (a scavenger of superoxide and hydroperoxyl radicals) has a strong influence decreasing significantly DBT conversion (see Figure S26).

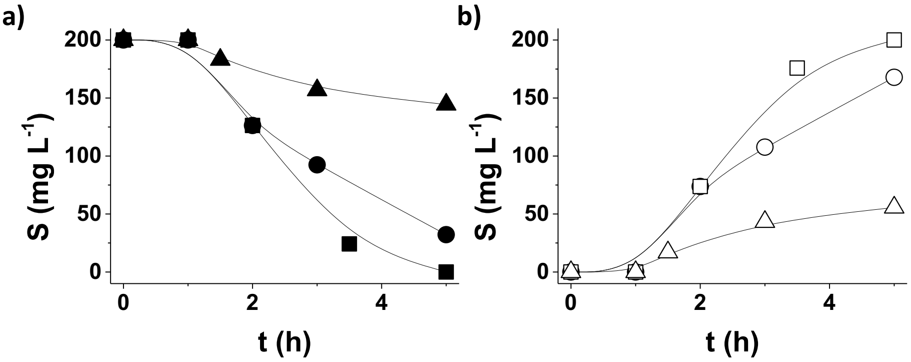

Fig. S26. Aerobic oxidation of (a) DBT to (b) $DBTO_2$ in the absence of any quencher (■), in the presence of DMSO (●) or in the presence of *p*-benzoquinone (▲). Reaction conditions: catalyst (5 mg of **MUV-2**), DBT (1150 mg L^{-1}), *n*-dodecane (10 mL), O_2 (1 atm), 140 ºC.

16.7. Raman spectra of MUV-2 with different atmospheres

Activation of molecular oxygen by $Fe_3\mu_3O$ nodes was assessed by *in situ* Raman spectroscopy. Upon activation of **MUV-2** at 220 ºC under N_2 and subsequent admission of O_2 at 140 ºC, appearance of two new vibration bands at 1502 and 1161 cm^{-1} attributable to adsorbed O_2 and Fe-O-O, superoxo, respectively, was observed (see Figure S27). These characteristic vibration bands disappear and the Raman spectra of the original thermally treated **MUV-2** Raman spectra is recovered by flushing N_2 on the sample, indicating that O_2 chemisorption was reversible.

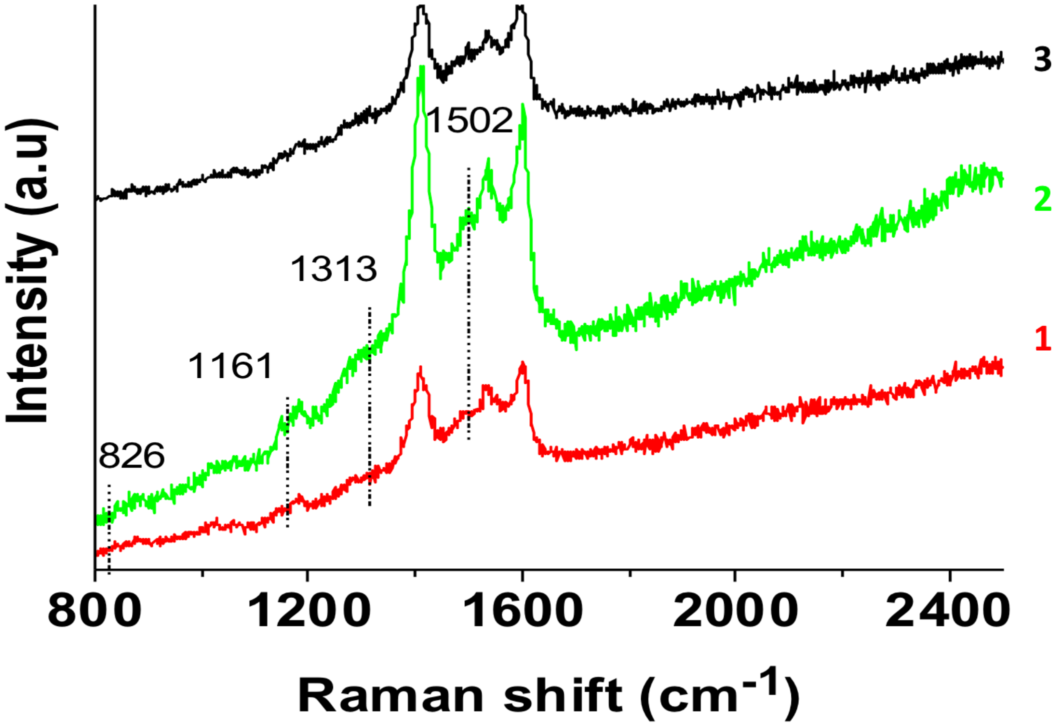

Fig. S27. (1) Raman spectra of **MUV-2** under N_2 flow at 140 ºC after being activated at 220 °C under N_2 flow for 2 h. (2) Same sample of spectrum 1 that is submitted subsequently to O_2 at 140 ºC for 2 h before recording the spectrum. (3) Sample of spectrum 2 that is submitted to N_2 at 140 ºC for 2 h before recording the spectrum. Legend: iron-oxo (826 cm^{-1}), iron-superoxo (1161 cm^{-1}) and adsorbed O2 (1313 and 1502 cm^{-1}).

16.8. Diesel as solvent

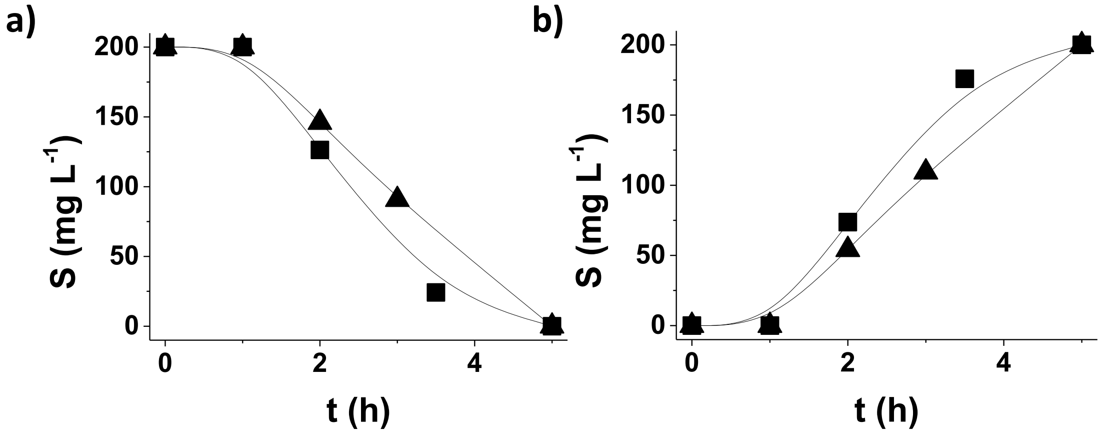

Fig. S28. Aerobic oxidation of DBT using **MUV-2** as catalyst (a) in *n*-dodecane (■) or diesel (▲) and (b) formation of the corresponding sulfone in *n*-dodecane (■) or in diesel (▲). Reaction conditions: catalyst (5 mg of **MUV-2**), DBT (1150 mg L^{-1}), *n*-dodecane or diesel (10 mL), O_2 (1 atm), 140 ºC.

17. References

1 Y. Mitamura, H. Yorimitsu, K. Oshima and A. Osuka, *Chem. Sci.*, 2011, **2**, 2017.
2 T. C. Narayan, T. Miyakai, S. Seki and M. Dincă, *J. Am. Chem. Soc.*, 2012, **134**, 12932–12935.
3 B. N. Figgis and G. B. Robertson, *Nature*, 1965, **205**, 694–693.
4 A. E. Earnshaw, B. N. Figgis and J. Lewis, *J. Chem. Soc.*, 1966, 1956.
5 D. Feng, K. Wang, Z. Wei, Y.-P. Chen, C. M. Simon, R. K. Arvapally, R. L. Martin, M. Bosch, T.-F. Liu, S. Fordham, D. Yuan, M. a Omary, M. Haranczyk, B. Smit and H.-C. Zhou, *Nat. Commun.*, 2014, **5**, 5723.
6 C. Serre, F. Millange, C. Thouvenot, M. Noguès, G. Marsolier, D. Louër and G. Férey, *J. Am. Chem. Soc.*, 2002, **124**, 13519–13526.
7 H. Nowell, S. A. Barnett, K. E. Christensen, S. J. Teat and D. R. Allan, *J. Synchrotron Radiat.*, 2012, **19**, 435–441.
8 G. M. Sheldrick, *Acta Crystallogr.*, 2015, **C71**, 3–8.
9 O. V. Dolomanov, L. J. Bourhis, R. J. Gildea, J. A. K. Howard and H. Puschmann, *J. Appl. Cryst.*, 2009, **42**, 339–341.
10 G. J. Long, T. E. Cranshaw and G. Longworth, *Mossb. Eff. Ref. Data J.*, 1983, **6**, 42–49.
11 J. C. Waerenborgh, P. Salamakha, O. Sologub, A. P. Gonçalves, C.Cardoso, S. Sério, M. Godinho and M. Almeida, *Chem. Mat.*, 2000, **12**, 1743–1749.
12 N. N. Greenwood and T. C. Gibb, *Mössbauer Spectroscopy*, Ltd. Publishers, London, 1971.
13 A. K. Boudalis, Y. Sanakis, C. P. Raptopoulou, A. Terzis, J.-P. Tuchagues and S. P. Perlepes, *Polyhedron*, 2005, **24**, 1540–1548.
14 M. François, M. I. Saleh, P. Rabu, M. Souhassou, B. Malaman and J. Steinmetz, *Solid State Sci.*, 2007, **7**, 1236–1246.
15 D. Piñero, P. Baran, R. Boca, R. Herchel, M. Klein, R. G. Raptis, F. Renz and Y. Sanakis, *Inorg. Chem.*, 2007, **46**, 10981–10989.
16 M. R. Silva, J. Coutinho, L. C. J. Pereira, P. Martín-Ramos and J. C. Waerenborgh, *Spectrochim. Acta A*, 2017, **172**, 9–13.
17 T. K. Hansen, T. Jørgensen, F. Jensen, P. H. Thygesen, K. Christiansen, M. B. Hursthouse, M. E. Harman, M. A. Malik, B. Girmay, A. E. Underhill, M. Begtrup, J. D. Kilburn, K. Belmore, P. Roepstorff and J. Becher, *J. Org. Chem.*, 1993, **58**, 1359–1366.
18 C. Wang, M. R. Bryce, A. S. Batsanov and J. A. K. Howard, *Chem. Eur. J.*, 1997, **3**, 1679–1690.
19 H.-Y. Wang, J.-Y. Ge, C. Hua, C.-Q. Jiao, Y. Wu, C. F. Leong, D. M. D'Alessandro, T. Liu and J.-L. Zuo, *Angew. Chemie Int. Ed.*, 2017, **56**, 5465 –5470.
20 S. Brunauer, P. H. Emmet and E. Teller, *J. Am. Chem. Soc.*, 1938, **69**, 309–319.
21 J. Rouquerol, P. Llewellyn and F. Rouquerol, *Stud. Surf. Sci. Catal.*, 2007, **160**, 49–56.
22 D. A. Cadenhead, J. F. Danielli and M. D. Rosenberg, *Prog. Surf. Membr. Sci.*, 1975, **9**, 1–70.
23 Y. H. Hu and E. Ruckenstein, *Chem. Phys. Lett.*, 2006, **425**, 306–310.
24 S. Ono, *J. Chem. Phys.*, 1950, **18**, 397–397.
25 T. L. Hill, *J. Chem. Phys.*, 1949, **17**, 520.
26 M. Palomino, A. Corma, F. Rey and S. Valencia, *Langmuir*, 2010, **26**, 1910–1917.
